# Effects of nutrient supply and nutrient ratio on diversity–productivity relationships of phytoplankton in the Cau Hai lagoon, Vietnam

**DOI:** 10.1002/ece3.5178

**Published:** 2019-04-25

**Authors:** Dang Thi Nhu Y, Nguyen Tien Hoang, Pham Khac Lieu, Hidenori Harada, Natacha Brion, Duong Van Hieu, Nguyen Van Hop, Harry Olde Venterink

**Affiliations:** ^1^ Department of Biology Vrije Universiteit Brussel (VUB) Brussels Belgium; ^2^ Department of Environmental Science, Hue College of Sciences Hue University Hue Vietnam; ^3^ Laboratory of Environmental Geosphere Engineering, Department of Urban Management, Graduate School of Engineering Kyoto University Kyoto Japan; ^4^ Department of Science, Technology and Environment Hue University Hue Vietnam; ^5^ Graduate School of Global Environmental Studies Kyoto University Kyoto Japan; ^6^ Analytical, Environmental and Geochemistry Vrije Universiteit Brussel (VUB) Brussels Belgium; ^7^ Department of Chemistry, Hue College of Sciences Hue University Hue Vietnam

**Keywords:** abundance, functional groups, phytoplankton, resource availability, resource ratio, richness

## Abstract

Diversity and productivity of primary producers are known to be influenced simultaneously by resource availability and resource ratio, but the relative importance of these two factors differed among studies and so far only entire phytoplankton communities were investigated which might ignore specific nutrient requirements and stoichiometric plasticity of different functional groups. We measured nutrient availability (DIN, total N [TN], total P [TP]), nutrient imbalance (TN:TP, DIN:TP, N:P_seston_), species richness, and abundance of the whole phytoplankton community, as well as those specific for cyanobacteria, diatoms, and dinoflagellates in Cau Hai lagoon in Vietnam. We determined the correlation among these variables, using structural equation modeling. The models applied to the whole phytoplankton community indicated that the nutrient availability (particularly TP and DIN) drove variation in phytoplankton abundance and richness, and that abundance also depended on species richness. The models applied to different functional groups differed considerably from the entire community and among each other, and only a part of the models was significant. The relationship between nutrient availability (mainly TP) and abundance was driven by cyanobacteria, and the relationship between nutrient imbalance (only with N:P_seston_) and species richness was driven by diatoms. Remarkably, the positive relationship between species richness and abundance, as consistently observed for the whole phytoplankton community, was only observed for one of the three functional groups (diatoms), indicating that resource complementarity occurs particularly among species of different functional groups. Our results emphasized that nutrient availability (TP and to a lesser extent DIN) as well as nutrient imbalance (albeit only with N:P_seston_ as proxy) were driving factors for the phytoplankton community in the Cau Hai lagoon and hence alterations in both of these factors leading to a shift in phytoplankton species composition and productivity.

## 1 INTRODUCTION

**Figure 1:**
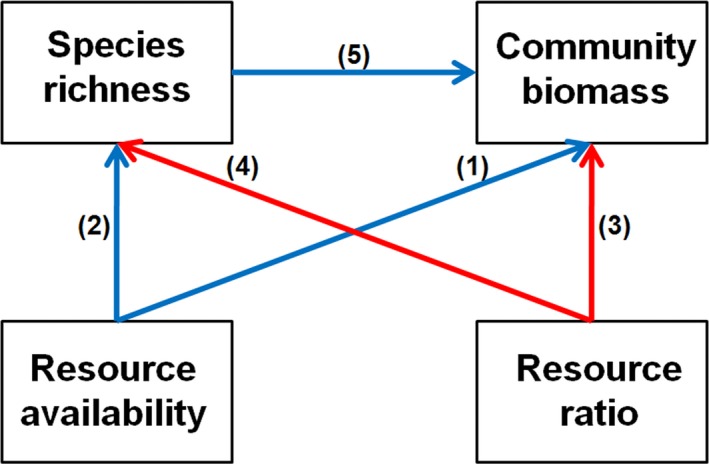
Hypothesized relationships among species richness, biomass, resource availability, and resource ratio according to Cardinale et al. (2009). Arrow numbers are explained in the text. Blue lines indicate positive paths, and red lines indicate negative paths

There is growing evidence that productivity–diversity relationships are influenced by both resource availability and resource ratio. In 2009, Cardinale et al. presented a conceptual model illustrating how these two nutrient factors influence the diversity and productivity of primary producers in aquatic ecosystems and supported their predictions with phytoplankton data from Norwegian freshwater lakes. The relationships among species richness, abundance, resource availability, and resource ratio as conceptualized by Cardinale, Hillebrand, Harpole, Gross, and Ptacnik (2009) (Figure 1) and supported by other previous and later studies are explained by the following mechanisms:

1. Community biomass is positively related to resource availability, since by definition community biomass increases with the availability of the limiting resource—which is often nitrogen or phosphorus depending on the type of ecosystem and local conditions (Cardinale et al., 2009; Elser et al., 2007; Korhonen, Wang, & Soininen, 2011; Lehtinen, Tamminen, Ptacnik, & Andersen, 2017; Lewandowska et al., 2016; Ptacnik et al., 2008; Smith, Joye, & Howarth, 2006; Vallina et al., 2014).
2. Species richness increases with nutrient availability because in line with the species–energy theory (SET), population sizes of resident species increase with nutrient availability, which reduces the risk of extinction of rare species (Cardinale et al., 2009; Wright, 1983). This positive relationship was supported for Norwegian and Finnish lakes (Cardinale et al., 2009; Korhonen et al., 2011), German and Finnish coastal waters (Hodapp, Meier, Muijsers, Badewien, & Hillebrand, 2015; Lehtinen et al., 2017) and a meta‐analysis from freshwater studies (Lewandowska et al., 2016). However, the relation with nutrient availability depends on the trophic state of aquatic ecosystems and varies from a positive linear unimodal relation in ultraoligotrophic systems to a negative linear relation in eutrophic systems (Korhonen et al., 2011).
3. Community biomass production is influenced by the ratio of nutrients because, according to the resource ratio theory (RRT), an imbalance of nutrients causes nutrient deficiency and reduces biomass production (Cardinale et al., 2009; Harpole & Tilman, 2007). Since a deficiency of both N and P can reduce productivity, the pattern of productivity with the N:P ratio is unimodal, as for instance demonstrated for plants (Olde Venterink & Güsewell, 2010). Therefore, whether productivity increases or decreases with N:P ratio depends on the range of N:P as well as on the trophic state of the ecosystem (Dolman & Wiedner, 2015; Lewandowska et al., 2016; Lv, Wun, & Chen, 2011; Pełechata, Pełechaty, & Pukacz, 2006). Cardinale et al. (2009) observed a negative relationship between biomass and N:P ratio in Norwegian lakes. In Finland, Lehtinen et al. (2017) also found a negative correlation between these factors for coastal waters, while Korhonen et al. (2011) observed a positive correlation for freshwater lakes.
4. Species richness is also related to nutrient ratios because, according to the R* hypothesis and the RRT, imbalance of nutrients leads to the exclusion of poor competitors for the most limiting nutrient which in turn reduce coexistence of species (Cardinale et al., 2009; Hillebrand, Cowlesb, Lewandowska, Waald, & Pluma, 2014; Tilman, 1982). Again, whether species richness increases or decreases with the N:P ratio depends on the range of N:P in the ecosystem (since it is a unimodal pattern). For Norwegian lakes and the Baltic Sea, a negative correlation was found for species richness and N:P ratio (Cardinale et al., 2009; Ganguly et al., 2015; Ptacnik, Andersen, & Tamminen, 2010), whereas a positive correlation was found for Finnish freshwaters and coastal waters (Korhonen et al., 2011; Lehtinen et al., 2017).
5. Biomass is positively related to species richness because of complementary resource use (Cardinale et al., 2009) of the most limiting nutrient resources, typically either nitrogen (N) or phosphorus (P) as frequently observed in aquatic ecosystems (Korhonen et al., 2011; Lehtinen et al., 2017; Lewandowska et al., 2016; Tian, Zhang, Zhao, Zhang, & Huang, 2017; Vallina et al., 2014).

Cardinale et al. (2009) tested their model for Norwegian lakes and found that all the above hypothesized relationships were supported (Appendix S1A). However, similar studies in other freshwater and marine ecosystems did not find significant correlations for some of the hypothesized relationships or sometimes even opposite patterns (Appendix S1B–E). Hence, there is a need for additional verification of the Cardinale et al. (2009) concept in other areas.

In addition, to our knowledge, the Cardinale et al. (2009) concept has only been tested for entire communities of (mainly) aquatic primary producers so far. However, the phytoplankton community is composed of different functional groups of species, which might have different nutrient requirements and constraints (Reynolds, 2006). Although species within and among functional groups will to a large extent compete for the same limited set of resources, it remains to be evaluated whether the concept also applies to specific functional groups of phytoplankton. For example, it is debated whether P supply and N:P ratio in the water are both influential for productivity of cyanobacteria: Some studies found that both are important (Filstrup, Heathcote, Kendall, & Downing, 2016; Smith, 1983), whereas other studies found that only P supply matters (Downing, Watson, & McCauley, 2001; Trimbee & Prepas, 1987; Watson, McCauley, & Downing, 1997). This difference—perhaps due to the possibility of some of the cyanobacteria species to fix atmospheric N_2_—will likely reduce the influence of the relative importance of the N:P ratio (arrows 3 and 4 in Figure 1). For diatoms, it has been demonstrated that the N:P ratio is more important for their growth, abundance, and diversity (arrows 3 and 4 in Figure 1) than P or N availability (arrows 1 and 2 in Figure 1; Guo et al., 2014). Hence, for this group of species, N:P arrows (3 and 4) may be more important than the resource availability arrows (1 and 2), at least in comparison with the entire phytoplankton community. Finally, the importance of both the N:P ratio and the resource availability arrows (arrows 3, 4 and 1, 2 in Figure 1, respectively) might be less important for dinoflagellates in comparison with those of the entire phytoplankton community, when resource availability and ratio are measured in the surface water. This is because dinoflagellates have mixotrophic and vertical migration abilities to explore resources (Hall & Paerl, 2011; Lin, Litaker, & Sunda, 2016).

In this study, we applied the concept of Cardinale et al. (2009) to the phytoplankton community of a brackish tropical lagoon system, the Cau Hai lagoon in Vietnam, in order to evaluate to which extent this general concept can explain the spatial variation in richness and abundance of phytoplankton. Thereto, we used combinations of TN and TP or DIN and TP as proxies for nutrient availability, as well as TN:TP, DIN:TP or the N:P ratio in seston as proxies for the ratio of nutrients. In order to highlight eventual differences between the overall phytoplankton community and different functional groups of species, we also applied this concept to cyanobacteria, diatoms, and dinoflagellates, separately.

For the whole phytoplankton community, we hypothesized that correlations between resource availability, resource ratio, species richness, and abundance would be in line with the concept of Cardinale et al. (2009), as illustrated in Figure 1. For cyanobacteria, we expected that the resource ratio arrows (3 and 4) would be less important compared with those in the overall phytoplankton community, and for diatoms that these arrows would be more important in comparison with the overall phytoplankton. For dinoflagellates, we expected both N:P ratio and resource availability arrows (1, 2, 3 and 4 in Figure 1) to be less important than those in the overall phytoplankton community. In addition to the Cardinale et al. (2009) approach, for which structural equation modeling is required, we also applied stepwise multiple regression with a larger set of environmental variables to explain variation in species richness and abundance of phytoplankton.

## 2 METHODS

### 2.1 Study site

**Figure 2:**
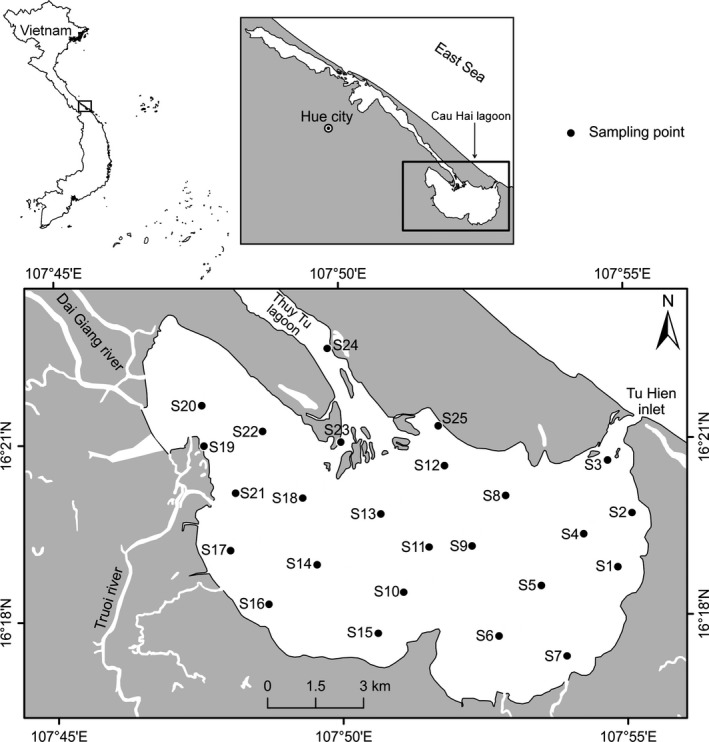
Location of sampling points for water, seston, and phytoplankton in the Cau Hai lagoon, central Vietnam. The black dots indicate the 25 sampling sites

The Cau Hai lagoon (16°19′22″N, 107°50′59″E) is a coastal lagoon, forming the Southern part of the Tam Giang–Cau Hai lagoon complex in the coastline of Thua Thien Hue province, Vietnam (Andrachuk, 2017; Figure 2). It covers a surface area of approximately 11,200 ha, with an average depth of 1.5 m (0.3–2.3 m) and is submitted to a microtidal regime (0.5–1.0 m; Truong, 2012). The climate of the region is typical tropical monsoon with a dry season lasting from May to September and a rainy season from October to April (Dang et al., 2015; Truong, 2012). The average water temperature during the sampling period varied between 25 and 35°C. Salinity ranged from 3 to 29‰ with temporal and spatial differences within the lagoon due to rainfall, river, and marine flows. Freshwater flows from the western part of the lagoon are considered as carriers of agricultural runoff and residential waste (Andrachuk, 2017). The lagoon is connected to the Tonkin Gulf through Tu Hien inlet.

Brackish water conditions create a variety of habitats and aquatic communities which support fishery and aquaculture (Disperati & Virdis, 2015; Nguyen & Yabe, 2014). The lagoon water quality is affected by nutrient enrichment from aquaculture, agricultural, and/or terrestrial runoff, which has increased the risk of eutrophication (Andrachuk, 2017; Nguyen & Yabe, 2014).

### 2.2 Sampling and measurements

Surface water, phytoplankton, and seston samples were simultaneously collected in March, May, and July 2016 at 25 sites in Cau Hai lagoon (located with Garmin GPS MAP^®^ 78; Figure 2). All glassware and containers were cleaned with a 5% H_2_SO_4_ solution and distilled water prior to use. All samples were placed in ice boxes after collection and transported to the laboratory. The details of sampling and measurements are as follows:

#### 2.2.1 Water

During each sampling event, in‐situ measurements were done about 30 cm below the water surface for water temperature, turbidity, and salinity, using a water quality monitoring unit with specific sensors (HORIBA U–5000). Surface water samples were collected with a Van Dorn sampler at 50 cm below the surface. The water samples were transferred into glass bottles and transported to the laboratory in an icebox. Samples for chlorophyll a (Chl*a*) analysis were obtained by filtration on glass fiber filters (Whatman GF/F) and stored in the dark at −20°C until analyses. Unfiltered water samples were stored in glass bottles and frozen until they were submitted to a digestion procedure for total N (TN) and total P (TP) analyses. Filtered samples were stored in glass bottles and frozen until they were analyzed for nitrate + nitrite (NO*_x_*) and ammonium (${\text{NH}}_{4}^{+}$) spectrophotometrically. All nutrient measurements followed standard protocols (APHA: American Public Health Association, 1999). The dissolved inorganic nitrogen (DIN) was calculated by summation of NO*_x_*–N and NH_4_–N. Trophic state indices (TSI) of the water were calculated using equations described by Carlson (1977), Kratzer and Brezonik (1981) for Chl*a*, TN, and TP, respectively.

#### 2.2.2 Phytoplankton

At each site, 2.0 L of surface water sample was collected and preserved with Lugol's solution buffered with formaldehyde (APHA: American Public Health Association, 1999), concentrated to 30 ml after sedimentation for 48 hr (Lv et al., 2011) and stored in brown glass bottles. Phytoplankton species were observed at the 400–1,000 × magnification (Olympus BX51) and morphologically identified using standard references (An, 1993; Fukuyo, Takano, Chihara, & Matsuoka, 1990; Tomas, 1997). Species richness was determined as the number of species at each site. The Sedgewick rafter counting cell slide was used for the enumeration of phytoplankton calculated as the number of cells/ml (McAlice, 1971; Park et al., 2018). We used this measurement of cell density as a proxy for abundance in this study.

#### 2.2.3 Seston

Surface water was filtered onto precombusted (450°C, 5 hr) Whatman GF/F filters (with a pore size of 0.7 µm) to collect seston (suspended particulate organic matter including detritus and living organisms) and then dried at 50°C for 24 hr. Prior to analysis, filters were fumed with HCl (37%, for 24 hr) in order to remove inorganic carbon (Meyer et al., 2016). After drying, filters were wrapped in tin cups and analyzed for C, N, and δ^15^N using an elemental analyzer (Flash1120 series EA; Thermo) coupled to an isotope ratio mass spectrometer (DeltaV; Thermo). Analyses were calibrated against reference materials: IAEA–CH6 (C_12_H_22_O_11_) and IAEA–N2 ((NH_4_)_2_SO_4_) were used for C and N respectively. Samples' isotopic ratios (*R*) was reported in the standard delta notation (*δ*) of the heavy to the light isotope (^15^N/^14^N in either sample or reference material) as follows: *δ* (‰) = [(*R*_Sample_/*R*_Standard_) − 1] × 1,000, with atmospheric N_2_ as the standard (West, Bowen, Cerling, & Ehleringer, 2006). TP was determined after combustion of the samples at 500°C for 2 hr and dissolving the residual in 1 M HCl (Andersen, 1976). Subsequently, phosphate was measured spectrophotometrically using a molybdate/ascorbic acid procedure with an automatic segmented flow nutrient analyzer (QuAAtro, Seal Analytical). N:P ratios of water column and C:N:P elemental ratios of seston were compared with the Redfield Ratio of 106:16:1 (Redfield, 1958) to evaluate potential growth‐limiting factors.

### 2.3 Data analysis

Linear regressions were used to determine the (correlative) influence of resource availabilities (DIN, TP) and resource ratio (N:P in seston) on abundance and species richness of total phytoplankton community and functional groups. Additionally, we used stepwise multiple regression with a larger set of explanatory variables (with time as a random factor) to evaluate the relative importance of nutrients (TN, DIN, TP, TN:TP, DIN:TP, N:P_seston_) compared with other environmental factors (temperature, turbidity, and salinity; Appendix S2).

Path analysis—a simple type of structural equation modeling (SEM; e.g., Grace, 2006; Cardinale et al., 2009)—was used to evaluate the effects of resource availability and resource ratio on the richness–biomass relationship of phytoplankton communities. We followed the classical approach of Cardinale et al. (2009) using their equations 2 and 4 to calculate resource availability (a) and resource ratios (*θ*), with either TN and TP as variables (following Cardinale et al., 2009, Korhonen et al., 2011) or with DIN and TP (following Lehtinen et al., 2017). Additionally, we calculated SEM models with DIN and TP in the water as proxy for resource availability (calculated as in Cardinale et al., 2009) and N:P in sestonas a proxy for resource balance. The latter follows Redfields classical observation that the balance between N and P can be measured in both the water and in marine particulate matter (seston; Redfield, 1958; Sterner & Elser, 2002). Moreover, we were interested in these alternative SEM models with N:P_seston_as proxy for N:P imbalance, because N:P_seston_was included in the stepwise multiple regression and also showed significant linear correlations with species richness, whereas TN:TP or DIN:TP were or did not. We note that seston includes living and dead phytoplankton and other organisms, as well as inorganic floating particles in the water column, but not the dissolved nutrients. Hence, N:P_seston_ does not fully reflect the ratio of total N and P availabilities in the system, but it may be closer to the ratio of N and P availabilities for the living organisms as it to a large extent measured in them. The R scripts of all models are shown in Appendix S5.

Because we had three repeated sampling events per site, we performed a path model with “time” as a random factor within “site” to account for the temporal dependency of the data, using the *lavaan.survey* package for complex survey analysis (Oberski, 2016) and *semPlot* package (Epskamp, 2015). In our SEM and multiple regression statistics, we assumed that the richness and abundance of the phytoplankton community were predominantly a result of the environmental and biotic factors that stimulated or inhibited the growth of a common set of species that can basically reach everywhere in the lagoon. We however cannot rule out that other spatial processes than gradients in nutrients or environmental conditions might have played a role as well. Spatial patterns of the most important variables for this study were shown in Appendices S3 and S4, including maps and results of principal component analysis and clustering techniques.

Model fitting was performed using maximum likelihood estimation with robust standard errors and the evaluation of the SEM models was carried out according to the criteria of Hu and Bentler (1999) and Cardinale et al. (2009). For a significant model, *p*‐values of the chi‐square test had to be >0.05 (models should not be significantly different from our theoretical model). Furthermore, larger values of the comparative fit index (CFI ranges from 0 to 1) and smaller values of root mean square error of approximation (RMSEA ranges from 0 to 1) indicated a better model fit (Hu & Bentler, 1999). Detailed statistical output of the SEM models is presented in Appendix S5. Noteworthy, the SEM model for dinoflagellates was evaluated as a significant model based on chi‐square *p*‐values, as well as CFI and RMSEA criteria, although none of the separate relationship (arrows) in the model was significant. Such a remarkable model has been published before (*cf*. Lewandowska et al., 2016).

Analyses were carried out with R version 3.4.0 on RStudio (Crawley, 2007; Logan, 2010; R Core Team, 2018).

## 3 RESULTS

**Figure 3:**
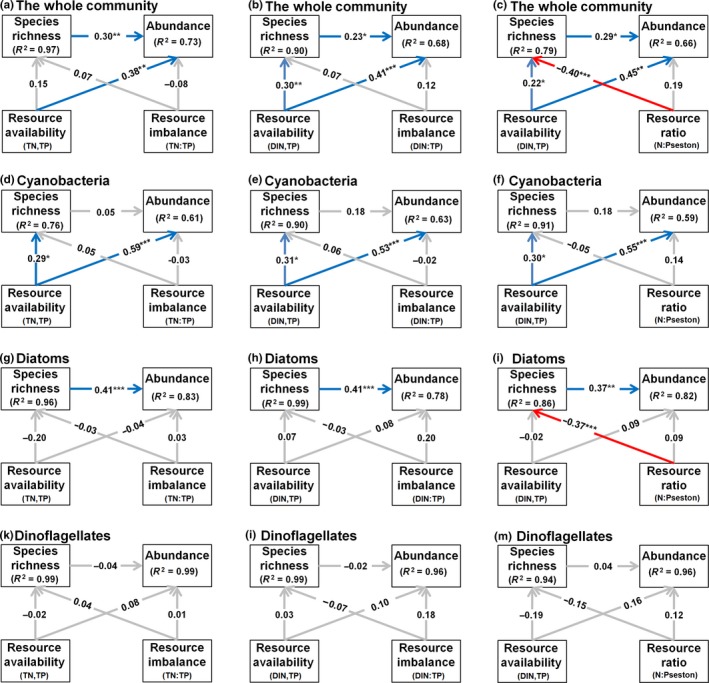
Direct and indirect effects of resource availability (TP, TN, DIN) and resource balance (TN:TP, DIN:TP or N:P_seston_) on the abundance and species richness of: (a–c) the whole phytoplankton communities (*n* = 75); (d–f) Cyanobacteria (*n* = 50); (g–i) Diatoms (*n* = 74), and (k–m) Dinoflagellates (*n* = 74). Blue lines indicate positive paths, and red lines indicate negative paths. *R* ^2^ values indicate the variance explained by the model for the various variables. Values on arrows are standardized path coefficients (equivalent to correlation coefficients) (*p < 0.05; **p < 0.01; ***p < 0.001). All models were significant. Full statistical output of the SEMs is shown in Appendix S5

Abundance of the whole phytoplankton community increased with resource availability in all SEM models (Figure 3a–c), whereas richness also increased with it when DIN + TP were used as proxy for availability (Figure 3b–c). We also found a significantly positive correlation between richness and abundance in all models (Figure 3a–c). Resource ratio only had an effect (negative) on species richness in the model with N:P_seston_ (Figure 3c). There was no significant correlation between resource ratio and abundance in any of the models (Figure 3a–c).

**Figure 4:**
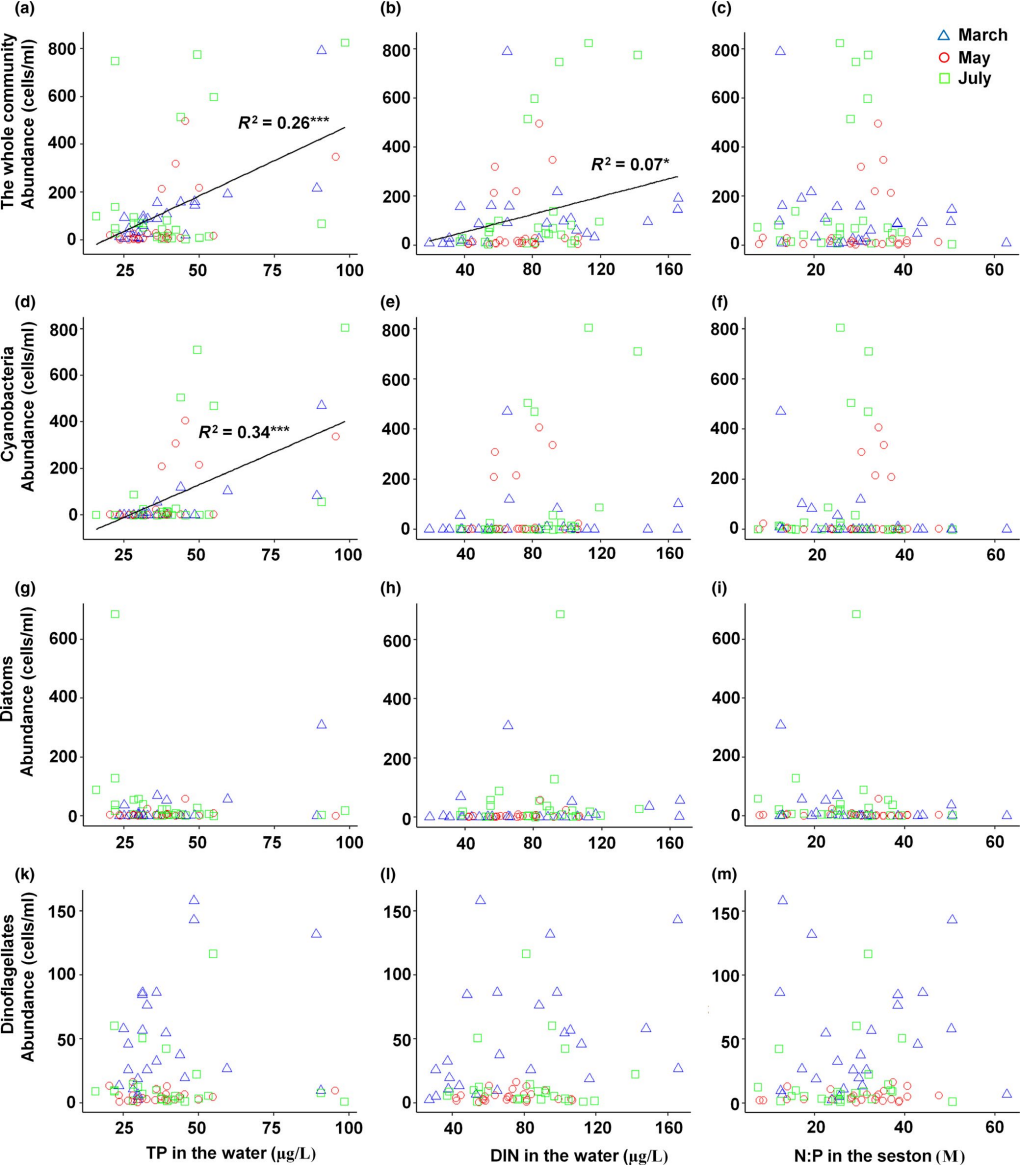
Phytoplankton abundance versus TP and DIN concentrations in water and N:P in seston in the Cau Hai lagoon in March, May, and July in 2016. Only significant regression lines are drawn (*p < 0.05; ***p < 0.001)

Relationships among species richness, abundance, resource availability, and resource ratio varied largely among phytoplankton groups (Figure 3d–m), but only in one case among models (Figure 3i compared 3g,h). For cyanobacteria, both species richness and abundance were positively correlated with resource availability in the water (Figure 4d–f). Resource ratio had only an effect on the diatom species richness, and only whether N:P_seston_ was used as proxy for the ratio (Figure 3i). A significant correlation between richness and abundance was observed for diatoms in all models (Figure 3g–i). None of the predicted paths were significant for the dinoflagellates community despite that the overall models were significant (Figure 3k–m).

The results of the multiple regression showed that variation in the abundance of the overall phytoplankton community was best explained by variations in TP and to a lesser extent by DIN and by salinity (Table 1). Variation in the abundance of cyanobacteria was best explained by variation in TP, that of diatoms by N:P_seston_, and that of dinoflagellates by temperature and, to a lesser extent, DIN in the water. Variation in species richness of the entire community was best explained by variation in N:P_seston_ and to lesser extent by turbidity of the water. Variation in richness of cyanobacteria species was best explained by TP in the water and by salinity, that of diatoms by turbidity, N:P_seston_, salinity, and temperature and that of dinoflagellates by N:P_seston_.

**Table 1:** Results of multiple regression analysis (with fixed site and time effect) for explaining variation in phytoplankton abundance and species richness

Dependent variables		Independent variables	Estimate	*SE*	*df*	*t*	*p*	Accumulated *R* ^2^
Abundance								
The whole community (*n* = 75)	Model 1	TP	2.34	0.35	49	6.75	<0.001	0.30
	Model 2	TP	1.99	0.32	48	0.24	<0.001	0.40
		DIN	0.94	0.24		3.91	<0.001	
	Model 3	TP	0.19	0.30	47	6.38	<0.001	0.46
		DIN	0.76	0.23		3.28	<0.01	
		Salinity	−0.97	0.32		−3.02	<0.01	
Cyanobacteria (*n* = 50)	Model 1	TP	2.90	0.55	26	5.28	<0.001	0.47
Diatoms (*n* = 74)	Model 1	N:P_seston_	−0.98	0.32	48	−3.01	<0.01	0.21
Dinoflagellates (*n* = 74)	Model 1	Temperature	−5.89	1.40	48	−4.20	<0.001	0.30
	Model 2	Temperature	−6.63	1.30	47	−5.09	<0.001	0.36
		DIN	0.79	0.28		2.86	<0.01	
Species richness								
The whole community (*n* = 75)	Model 1	N:P_seston_	−0.01	0.01	49	−3.89	<0.001	0.28
	Model 2	N:P_seston_	−0.01	0.01	48	−3.90	<0.001	0.33
		Turbidity	0.10	0.04		2.51	<0.05	
Cyanobacteria (*n* = 50)	Model 1	TP	0.59	0.13	27	4.54	<0.001	0.34
	Model 2	TP	0.43	0.14	25	3.03	<0.01	0.40
		Salinity	−0.35	0.16		−2.28	<0.05	
Diatoms (*n* = 74)	Model 1	Turbidity	0.22	0.05	48	4.11	<0.001	0.20
	Model 2	Turbidity	0.19	0.05	47	3.54	<0.001	0.28
		N:P_seston_	−0.41	0.13	47	−3.20	<0.01	
	Model 3	Turbidity	0.27	0.07	45	3.54	<0.001	0.36
		N:P_seston_	−0.39	0.12		−3.19	<0.01	
		Salinity	0.42	0.15		2.74	<0.01	
		Temperature	−1.64	0.77		−2.13	<0.05	
Dinoflagellates (*n* = 74)	Model 1	N:P_seston_	−0.21	0.09	48	−2.25	<0.05	0.10

Note

Predictor variables were TP, TN, DIN, TP:TN, DIN:TP, N:P_seston_, water temperature, salinity, and turbidity.

**Figure 5:**
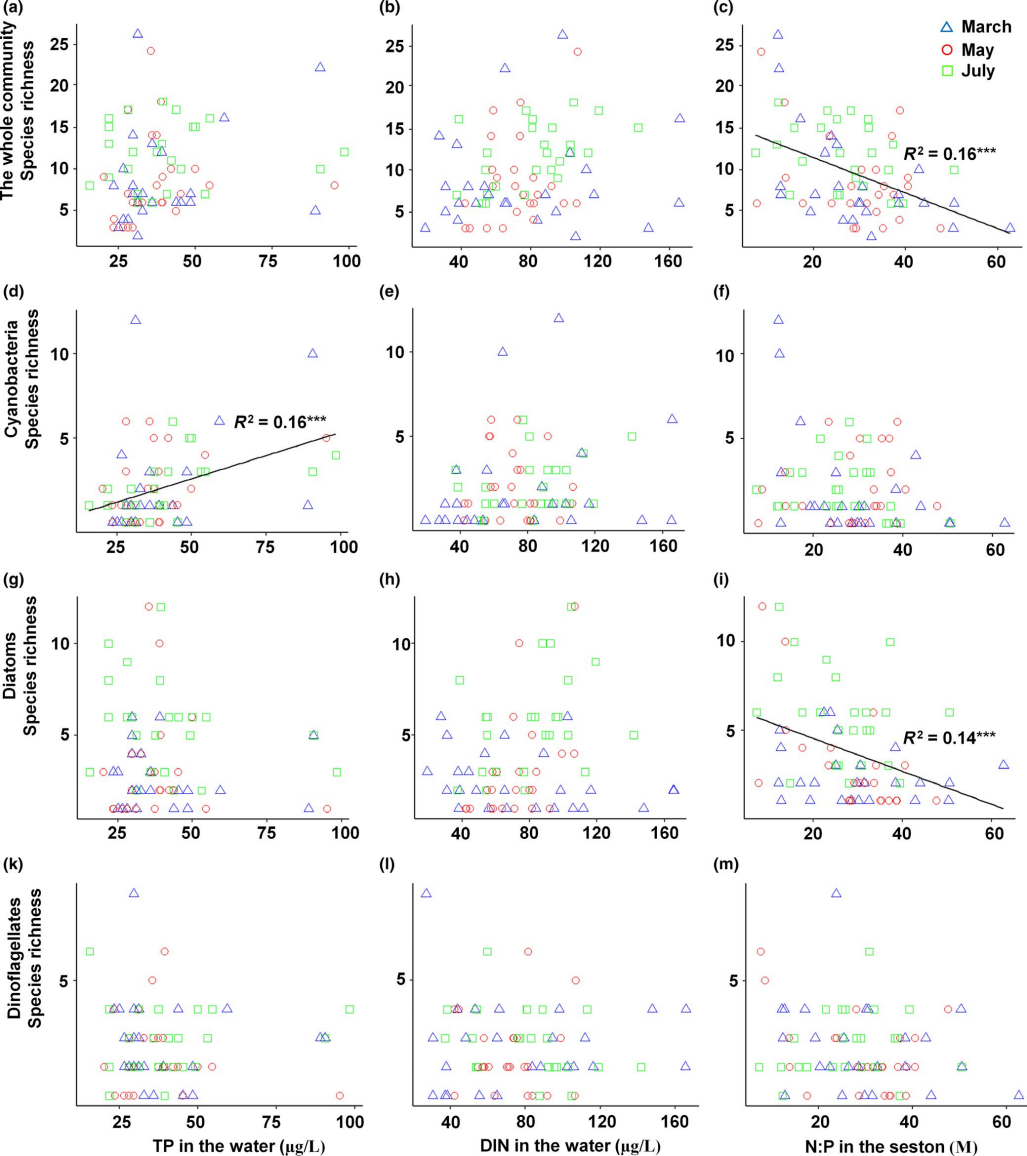
Phytoplankton species richness versus TP and DIN concentrations in water and N:P in seston in the Cau Hai lagoon in March, May, and July in 2016. Only significant regression lines are drawn (***p < 0.001)

The positive correlations between TP and DIN in water with the abundance of the phytoplankton community and for TP with cyanobacteria abundance were also found by linear regressions (Figure 4). Species richness of the entire community as well as a diatom richness was negatively correlated with N:P_seston_ and that of cyanobacteria positively with TP in the water (Figure 5). Neither abundance nor richness of the whole community or any of the functional groups was significantly correlated with ratios of TN:TP or DIN:TP.

## 4 DISCUSSION

### 4.1 The Cardinale concept applied to the whole phytoplankton community

The concept of Cardinale et al. (2009) was supported for 2–4 out of 5 arrows in the Cau Hai lagoon, depending on the proxies used for nutrient availability or ratio (Figure 3a–c). The correlations between resource availability and abundance and species richness of phytoplankton (arrows 1 and 2), between resource ratio and species richness (arrow 4), as well as between species richness and abundance (arrow 5), were all in line with our hypotheses for at least one of our SEM models (i.e., Figure 3a, b or c), and as such support the underlying mechanistic assumptions as mentioned in the Introduction. Firstly, community abundance increased with increasing TP + DIN or TP + TN concentrations in the water (arrow 1). Moreover, the results of the multiple regression (Table 1) and of simple linear regressions (Figure 4a,b) indicated that TP was the main growth‐limiting nutrient in the Cau Hai lagoon, which was consistent with several other tropical coastal waters (Elser et al., 2007; Fourqurean, Manuel, Coates, Kenworthy, & Boyer, 2015). Secondly, species richness of the whole phytoplankton community increased with increasing nutrient availability if TP + DIN were used as a proxy (arrow 2 in Figure 3b,c, but not 3a), which was in line with the species–energy theory (Cardinale et al., 2009; Wright, 1983) and with various previous studies in lakes and coastal waters (Cardinale et al., 2009; Hodapp et al., 2015; Korhonen et al., 2011; Lehtinen et al., 2017; Lewandowska et al., 2016). Thirdly, species richness decreased with increasing ratio of N and P, albeit only if the N:P ratio in seston was used as a proxy (arrow 4 in Figure 3c; Figure 5c). The ratios of N:P_Seston_(29 ± 1) and C:P_Seston_ (208 ± 8; Appendix S6) were far above the Redfield ratio (16 and 106 respectively; Redfield, 1958), which again illustrated that P rather than N or C fixation was the limiting factor for the growth of the phytoplankton community in the Cau Hai lagoon. Hence, species exclusion because of resource imbalance in the Cau Hai lagoon was also most severe under the most extreme P‐limited conditions (following the predicted mechanism behind arrow 4), although this was not observed when imbalances in TN:TP or DIN:TP in the water were used in the models (Figure 3a,b; see below). A negative correlation between species richness and N:P ratio in the water was also observed in the East China Sea (Guo et al., 2014), although the authors ascribed this pattern to indirect effect of community abundance. Fourthly, as predicted, community abundance increased with species richness, supporting the mechanism of complementary resource use (arrow 5). In this respect, our results agreed with Tian et al. (2017) who concluded that positive effects of richness on biomass are common in oligotrophic to slightly eutrophic lakes. The Cau Hai lagoon could be considered as mesotrophic (average TSI = 46 ± 1, Appendix S6) and hence fitted in this range.

In contrast to the hypothesized model, no significant pattern was found for the effect of resource ratio on the abundance of the phytoplankton community in the Cau Hai Lagoon (arrow 3 in Figure 3a–c). We also did not observe a significant linear (or unimodal) correlation between abundance and N:P in seston (Figure 4c), nor between abundance and TN:TP or DIN:TP in water (data not shown). Hence, our results were not in line with the resource ratio theory (*cf*. Harpole & Tilman, 2007; Cardinale et al., 2009). Lewandowska et al., (2016) used N:P imbalance in their meta‐analysis and found a positive rather than a negative relationship for arrow 3 for marine ecosystems and no significant pattern for freshwater ecosystems (Appendix S1). They ascribed the discrepancy of these outcomes (compared with the theoretical predictions of Cardinale et al., 2009) to the limited number of resources included in the model (only N and P, Lewandowska et al., 2016).

The concept of Cardinale et al. (2009) has been applied in several aquatic ecosystems (see overview in Appendix S1), but apart from the Norwegian lakes of the Cardinale et al. (2009) study itself, the model was never fully supported in any of the other areas. Our results for the Cau Hai lagoon yielded two, three, or four significant paths (always in the predicted direction) depending on the proxies used for nutrient availability or nutrient balance. Only the effect of resource imbalance on community abundance was not significant in our models (Figure 3a–c). The classical Cardinale model with TN and TP only yielded two significant paths. Using DIN instead of TN for nutrient availability showed a significant effect of nutrient availability on species richness. This may either reflect that DIN in water was a more direct measure of nutrient availability than TN, which includes both N availability (DIN) as well as the response to it (N taken up by organisms), or it may illustrate that the richness of different functional groups (cyanobacteria and diatoms) reflected oppositely to TN but not to DIN (Figure 3d,g). Apart from the results of the meta‐analysis for marine studies of Lewandowska et al. (2016), the positive effects of resource availability on abundance and richness are in line with observations in similar other studies (Appendix S1).

The effect of nutrient imbalance was only significant if N:P in seston was used in the SEM model, and not when the imbalance between TN:TP or DIN:TP was used (Figure 3a–c). This may be due to an intrinsic difference between N:P ratio or imbalance as calculated according to the method of Cardinale et al. (2009), or it may show that N:P in seston reflected the imbalance between N and P availabilities during the growing season better than the balance of N and P in the water at a certain moment. Concentrations of mineral N and P in water vary in time and autotrophs tend to accumulate the nonlimiting elements in their tissue (Marschner, 2012), which might enlarge and emphasize the variation in N:P imbalance among sites stronger than the ratio of mineral N and P forms in water. Noteworthy, if N:P in seston would correlate with abundance rather than with richness of phytoplankton one should be careful with drawing conclusions, since the N:P in autotrophs decreases with increasing productivity according to the growth rate hypothesis (Sterner & Elser, 2002). In our study, N:P in seston was only correlated with the abundance of diatoms (Table 1), and it did not significantly explain abundance in the SEM models (Figure 3). There are considerable differences among studies in the effect of resource ratio or imbalance on species richness, with both positive and negative effects observed (Appendix S1; Cardinale et al., 2009; Korhonen et al., 2011; Lehtinen et al., 2017; Lewandowska et al., 2016). These differences are related to the range and the variation in N:P stoichiometry included in the studies sites. A negative correlation was found between species richness and N:P balance or ratio in Norwegian lakes and the Baltic sea (Cardinale et al., 2009; Ganguly et al., 2015; Ptacnik et al., 2010) and a positive correlation in freshwaters and coastal waters in Finland (Korhonen et al., 2011; Lehtinen et al., 2017). The negative correlation between species richness and N:P in seston in the Cau Hai lagoon (Figures 3c and 5c) pointed to competitive exclusion for P according to the R* and resource ratio theories, assuming that P is primary limiting resource which is consistent with the observed relationship between TP and phytoplankton abundance (Table 1).

Overall, the significant models of Figure 3a–c illustrated that TP and DIN availabilities and the N:P ratio were decisive factors for the phytoplankton community in the Cau Hai lagoon and hence alterations in both these factors leading to a shift in phytoplankton species composition and productivity.

### 4.2 The Cardinale concept applied to separate functional groups

For cyanobacteria, our hypothesis was supported in the sense that both richness and abundance were positively correlated with resource availability (TP + TN or TP + DIN), yielding significant positive arrows 1 and 2, but N:P balance or ratio had no significant effect (Figure 3d–f). The results of the multiple regression (Table 1) and the simple linear regressions (Figures 4d and 5d) indicated that particularly variations in TP concentration played and importance role in explaining cyanobacteria abundance and species richness. Hence, the results of the Cau Hai lagoon were in line with a group of studies that found that only P supply matters for cyanobacteria productivity and diversity (Downing et al., 2001; Trimbee & Prepas, 1987; Watson, Ridal, & Boyer, 2008) and not to another group of studies that found that besides P also N:P was important (Filstrup et al., 2016; Smith, 1983). We assumed that the N:P balance might be less important for the cyanobacteria than for the entire phytoplankton community because of the ability of some cyanobacteria to fix atmospheric N_2_. However, the relatively high δ^15^N in seston (on average 8.7 ± 3‰) suggests that N_2_fixation was not an important source of N for phytoplankton or cyanobacteria in the Cau Hai lagoon (Appendix S6). Instead, the N availability in the Cau Hai lagoon was generally high, at least compared with TP availability and the fast majority of the sites had an N:P >16 (Figures 4 and 5), which makes N availability and the N:P balance less important than P availability for variation in species richness and productivity. Noteworthy, we only observed significant effects of resource availability in our model for cyanobacteria and not for the other two functional groups, which indicated that cyanobacteria were more efficient in resource use than other phytoplankton taxa (Filstrup et al., 2016; Tian et al., 2017) and that particularly P favors their dominance (Smith, 1983).

We found some support for our hypothesis for diatoms: that is, we observed a significant correlation between species richness and N:P ratio (albeit it only with N:P_seston_) and not with P availability (Figure 3i). We also predicted that diatom abundance would be more affected by nutrient ratio than by nutrient availability, but this was not supported by the SEM models (Figure 3g–i). The multiple regression, however, also illustrated the importance of N:P_seston_ for diatom abundance, as well as N:P_seston_, turbidity, salinity, and temperature for diatom richness (Table 1). Our results were consistent with previous observations in marine environments showing that low N:P habitats are generally diatom‐rich and high N:P habitats are diatom‐poor (Galbraith & Martiny, 2015; Hillebrand et al., 2013; Redfield, 1958). It indicated that the N:P balance was a main driver for the numbers of coexisting diatom species (Galbraith & Martiny, 2015). A negative correlation between N:P_seston_ and diatom abundance was consistent with similar observations from the East China Sea (Guo et al., 2014). We also observed a positive effect of species richness on diatom abundance (Figure 3g–i), which points to resource complementarity within the diatom community. The observed correlation between species richness of diatoms and turbidity in the water column (Table 1) might be related to intense mixing of the water column and preventing dominance of a few species and hence higher species richness (Hodapp et al., 2015; Oliver, Mitrovic, & Rees, 2010).

Our hypothesis for dinoflagellates was also supported; we did not find any significant relationship in the dinoflagellates models (Figures 3k–m, 4k–m, and 5k–m). As expected, dinoflagellates appear to be far less dependent on nutrients in the surface water because their mobility and nutritional flexibility enable them to exploit the surface sediment beside the surface water (Hall & Paerl, 2011). The abundance of dinoflagellates was particularly correlated with temperature (negative correlation; Table 1). A high water temperature as observed in Cau Hai lagoon (range from 25–35°C; Appendix S6) might exceed optimal growth conditions for studied dinoflagellate species (generally below 30°C; Warner, Fitt, & Schmidt, 1999; Sparrow, Momigliano, Russ, & Heimann, 2017) and hence result in lower abundances with increasing water temperature.

### 4.3 Conclusions from applying the Cardinale et al. (2009) concept to different functional groups and the whole community in a tropical coastal lagoon

When comparing the models of the separate functional groups with that of the entire phytoplankton community in the Cau Hai lagoon, it appears that the positive relationship between abundance and resource availability (arrow 1) in the entire community was driven by variation in cyanobacteria abundance, whereas the negative relationship between species richness and N:P ratio (arrow 4) was driven by variation in diatom richness. Hence, variations within these functional groups which have significant contribution to the entire phytoplankton species richness and abundance (Appendix S7) were decisive for the entire phytoplankton community. Species richness appeared to be a very consistent driving factor for variation in abundance, both in previous studies (Appendix S1) and in our study in the Cau Hai lagoon, at least when the overall phytoplankton community was considered (arrows 5 in Figure 3a–c). Noteworthy, this relationship was not significant in the separate models for two out of three functional groups in the Cau Hai lagoon (Figure 3d–m). Likely, the variation in functionality of resource use was larger in the entire community than in separate functional groups, hence having a greater chance of resource complementarity in the entire community (*cf*. Cardinale et al., 2009).

## CONFLICT OF INTEREST

The authors declare no competing interests.

## AUTHOR CONTRIBUTIONS

D.T.N.Y. and H.O.V. designed the study; N.T.H made sampling map; D.T.N.Y. collected field samples and analyzed water, seston, and phytoplankton samples; N.B. assisted with chemical analyses. D.T.N.Y. and H.O.V. wrote the manuscript with contributions from all co‐authors.

## Data Availability

Network for Biocomplexity. https://doi.org/10.5063/F1J38QSG.

## References

[ece35178-bib-0001] An, T. N. (1993). Planktonic diatom in the East Sea, Vietnam. Publishing House of Science and Technology, 315 pp (in Vietnamese).

[ece35178-bib-0002] Andersen, J. M. (1976). An ignition method for determination of total phosphorus in lake sediments. Water Research, 10, 329–331.

[ece35178-bib-0003] Andrachuk, M. (2017). Exploring pathways for social‐ecological transformation in the Cau Hai lagoon, Vietnam, PhD Thesis. Waterloo, Ontario, 198 pp.

[ece35178-bib-0004] APHA: American Public Health Association (1999). Standard methods for the examination of water and wastewater, 20th ed Washington DC: American Public Health Association, American Water Works Association, Water Environment Federation.

[ece35178-bib-0005] Cardinale, B. J. , Hillebrand, H. , Harpole, W. S. , Gross, K. , & Ptacnik, R. (2009). Separating the influence of resource ‘availability' from resource ‘imbalance' on productivity–diversity relationships. Ecology Letters, 12, 475–487. 19490011 10.1111/j.1461-0248.2009.01317.x

[ece35178-bib-0006] Carlson, R. E. (1977). A trophic state index for lakes. Limnology and Oceanography, 22, 361–369.

[ece35178-bib-0007] Crawley, M. J. (2007). The R book. London, UK: Wiley, 950 pp.

[ece35178-bib-0008] Dang, H. N. , Nguyen, N. A. , Nguyen, D. K. , Bui, V. L. , Nguyen, V. Q. , & Phan, S. H. (2015). Sedimentation in Coastal lagoons: Tam Giang‐Cau Hai, Thi Nai and Nai in the Centre of Viet Nam. VNU Journal of Earth and Environmental Sciences, 31(3), 15–25. (in Vietnamese).

[ece35178-bib-0009] Disperati, L. , & Virdis, S. G. P. (2015). Assessment of land–use and land–cover changes from 1965 to 2014 in Tam Giang‐Cau Hai lagoon, Central Vietnam. Applied Geography, 58, 48–64.

[ece35178-bib-0010] Dolman, A. M. , & Wiedner, C. (2015). Predicting phytoplankton biomass and estimating critical N:P ratios with piecewise models that conform to Liebig's law of the minimum. Freshwater Biology, 60, 686–697.

[ece35178-bib-0011] Downing, J. A. , Watson, S. B. , & McCauley, E. (2001). Predicting Cyanobacteria dominance in lakes. Canadian Journal of Fisheries and Aquatic Science, 58, 1905–1908.

[ece35178-bib-0012] Elser, J. J. , Bracken, M. E. S. , Cleland, E. E. , Gruner, D. S. , Harpole, W. S. , Hillebrand, H. , … Smith, J. E. (2007). Global analysis of nitrogen and phosphorus limitation of primary producers in freshwater, marine and terrestrial ecosystems. Ecology Letters, 10, 1135–1142. 17922835 10.1111/j.1461-0248.2007.01113.x

[ece35178-bib-0013] Epskamp, S. (2015). semPlot: Unified visualizations of structural equation models. Structural Equation Modeling, 22, 474–483.

[ece35178-bib-0014] Filstrup, C. T. , Heathcote, A. J. , Kendall, D. L. , & Downing, J. A. (2016). Phytoplankton taxonomic compositional shifts across nutrient and light gradients in temperate lakes. Inland Waters, 6(2), 234–249.

[ece35178-bib-0015] Fourqurean, J. W. , Manuel, S. A. , Coates, K. A. , Kenworthy, W. J. , & Boyer, J. N. (2015). Water quality, isoscapes and stoichioscapes of seagrasses indicate general P limitation and unique N cycling in shallow water benthos of Bermuda. Biogeosciences, 12, 6235–6249.

[ece35178-bib-0016] Fukuyo, Y. , Takano, H. , Chihara, M. , & Matsuoka, K. (1990). Red tide organisms in Japan – An illustrated taxonomic guide. Tokyo, Japan: Uchida Rokakuho, 430 pp.

[ece35178-bib-0017] Galbraith, E. D. , & Martiny, A. C. (2015). A simple nutrient–dependence mechanism for predicting the stoichiometry of marine ecosystems. Proceedings of the National Academy of Sciences of the USA, 112(27), 8199–8204. 26056296 10.1073/pnas.1423917112 PMC4500256

[ece35178-bib-0018] Ganguly, D. , Patra, S. , Muduli, P. R. , Vardhan, K. V. , Abhilash, K. R. , Robin, R. S. , & Subramanian, B. R. (2015). Influence of nutrient input on the trophic state of a tropical brackish water lagoon. Journal of Earth System Science, 124, 1005–1017.

[ece35178-bib-0020] Grace, J. B. (2006). Structural equation modeling and natural systems. Cambridge, UK: Cambridge University Press, 365 pp.

[ece35178-bib-0021] Guo, S. , Feng, Y. , Wang, L. , Dai, M. , Liu, Z. , Bai, Y. , & Sun, J. (2014). Seasonal variation in the phytoplankton community of a continental–shelf sea: The East China Sea. Marine Ecology Progress Series, 516, 103–126.

[ece35178-bib-0022] Hall, N. S. , & Paerl, H. W. (2011). Vertical migration patterns of phytoflagellates in relation to light and nutrient availability in a shallow microtidal estuary. Marine Ecology Progress Series, 425, 1–9.

[ece35178-bib-0023] Harpole, W. S. , & Tilman, D. (2007). Grassland species loss resulting from reduced niche dimension. Nature, 466, 791–793. 10.1038/nature05684 17384633

[ece35178-bib-0024] Hillebrand, H. , Cowlesb, J. M. , Lewandowska, A. , Van de Waald, D. B. , & Pluma, C. (2014). Think ratio! A stoichiometric view on biodiversity–ecosystem functioning research. Basic and Applied Ecology, 15, 465–474.

[ece35178-bib-0025] Hillebrand, H. , Steinert, G. , Boersma, M. , Malzahn, A. , Meunier, C. L. , Plum, C. , & Ptacnik, R. (2013). Goldman revisited: Faster growing phytoplankton has lower N:P and lower stoichiometric flexibility. Limnology and Oceanography, 58, 2076–2088.

[ece35178-bib-0026] Hodapp, D. , Meier, S. , Muijsers, F. , Badewien, T. H. , & Hillebrand, H. (2015). Structural equation modeling approach to the diversity−productivity relationship of Wadden Sea phytoplankton. Marine Ecology Progress Series, 523, 31–40.

[ece35178-bib-0027] Hu, L. , & Bentler, P. M. (1999). Cutoff criteria for fit indexes in covariance structure analysis: Conventional criteria versus new alternatives. Structural Equation Modeling, 6, 1–55.

[ece35178-bib-0028] Korhonen, J. J. , Wang, J. , & Soininen, J. (2011). Productivity‐diversity relationships in lake plankton communities. PLoS ONE, 6(8), e22041 10.1371/journal.pone.0022041 21850218 PMC3151241

[ece35178-bib-0029] Kratzer, C. R. , & Brezonik, P. L. (1981). A carlson–type trophic state index for nitrogen in Florida lakes. Water Resources Bulletin, 17, 713–715.

[ece35178-bib-0030] Lehtinen, S. , Tamminen, T. , Ptacnik, R. , & Andersen, P. (2017). Phytoplankton species richness, evenness, and production in relation to nutrient availability and imbalance. Limnology and Oceanography, 62, 1393–1408.

[ece35178-bib-0031] Lewandowska, A. M. , Biermann, A. , Borer, E. T. , Cebrián‐Piqueras, M. A. , Declerck, S. A. J. , De Meester, L. , … Hillebrand, H. (2016). The influence of balanced and imbalanced resource supply on biodiversity–functioning relationship across ecosystems. Philosophical Transactions of the Royal Society B: Biological Sciences, 371, 20150283. 10.1098/rstb.2015.0283 PMC4843703 27114584

[ece35178-bib-0032] Lin, S. , Litaker, R. W. , & Sunda, W. G. (2016). Phosphorus physiological ecology and molecular mechanisms in marine phytoplankton. Journal of Phycology, 52, 10–36. 26987085 10.1111/jpy.12365

[ece35178-bib-0033] Logan, M. (2010). Biostatistical design and analysis using R: A practical guide. London, UK: Wiley & Blackwell, 546 pp.

[ece35178-bib-0034] Lv, J. , Wun, H. , & Chen, M. (2011). Effects of nitrogen and phosphorus on phytoplankton composition and biomass in 15 subtropical, urban shallow lakes in Wuhan, China. Limnologica, 41, 48–56.

[ece35178-bib-0035] Marschner, P. (2012). Mineral nutrition of higher plants. London, UK: Academic Press, 651 pp.

[ece35178-bib-0036] McAlice, B. J. (1971). Phytoplankton sampling with the Sedgewick Rafter Cell. Limnology and Oceanography, 16, 19–28.

[ece35178-bib-0037] Meyer, J. , Löscher, C. R. , Neulinger, S. C. , Reichel, A. F. , Loginova, A. , Borchard, C. , … Riebesell, U. (2016). Changing nutrient stoichiometry affects phytoplankton production, DOP accumulation and dinitrogen fixation–a mesocosm experiment in the eastern tropical North Atlantic. Biogeosciences, 13, 781–794.

[ece35178-bib-0038] Nguyen, Q. C. T. , & Yabe, M. (2014). Shrimp poly‐culture development and local livelihoods in Tam Giang‐Cau Hai lagoon. Vietnam. Agricultural Science, 6, 1–14.

[ece35178-bib-0039] Oberski, D. (2016). lavaan.survey: An R package for complex survey analysis of structural equation models. Journal of Statistical Software, 10(2), 1–24.

[ece35178-bib-0040] Olde Venterink, H. , & Güsewell, S. (2010). Competitive interactions between two meadow grasses under nitrogen and phosphorus limitation. Functional Ecology, 24, 877–886.

[ece35178-bib-0041] Oliver, R. L. , Mitrovic, S. M. , & Rees, C. (2010). Influence of salinity on light conditions and phytoplankton growth in a turbid river. River Research and Applications, 26, 894–903.

[ece35178-bib-0042] Park, H. K. , Byeon, M. S. , Choi, M. J. , Yun, S. H. , Jeon, N. H. , You, K. A. , & Lee, H. J. (2018). Water quality improvement through the interaction of biotic and abiotic variables within the rhizospheric zone of an artificial floating vegetation island. Freshwater Ecology, 33(1), 57–72.

[ece35178-bib-0043] Pełechata, A. , Pełechaty, M. , & Pukacz, A. (2006). An attempt to the trophic status assessment of the lakes of Lubuskie Lakeland. Limnological Review, 6, 239–246.

[ece35178-bib-0044] Ptacnik, R. , Andersen, T. , & Tamminen, T. (2010). Performance of the Redfield ratio and a family of nutrient limitation indicators as thresholds for phytoplankton N vs. P Limitation. Ecosystems, 13, 1201–1214.

[ece35178-bib-0045] Ptacnik, R. , Solimini, A. G. , Andersen, T. , Tamminen, T. , Brettum, P. , Lepisto, L. , … Rekolainen, S. (2008). Diversity predicts stability and resource use efficiency in natural phytoplankton communities. Proceedings of the National Academy of Sciences of the USA, 105, 5134–5138. 18375765 10.1073/pnas.0708328105 PMC2278227

[ece35178-bib-0046] R Core Team (2018). R: A language and environment for statistical computing. Vienna, Austria: R Foundation for Statistical Computing.

[ece35178-bib-0047] Redfield, A. C. (1958). The biological control of chemical factors in the environment. American Scientist, 46, 561–600.

[ece35178-bib-0048] Reynolds, C. S. (2006). The ecology of phytoplankton. New York, NY: Cambridge University Press, 535 pp.

[ece35178-bib-0049] Smith, V. H. (1983). Low nitrogen to phosphorus ratios favor dominance by blue–green algae in lake phytoplankton. Science, 221, 669–671. 17787737 10.1126/science.221.4611.669

[ece35178-bib-0050] Smith, V. H. , Joye, S. B. , & Howarth, R. W. (2006). Eutrophication of freshwater and marine ecosystems. Limnology and Oceanography, 51, 351–355.

[ece35178-bib-0051] Sparrow, L. , Momigliano, P. , Russ, G. R. , & Heimann, K. (2017). Effects of temperature, salinity and composition of the dinoflagellate assemblage on the growth of Gambierdiscus carpenter isolated from the Great barrier Reef. Harmful Algae, 65, 52–60. 28526119 10.1016/j.hal.2017.04.006

[ece35178-bib-0052] Sterner, R. W. , & Elser, J. J. (2002). Ecological stoichiometry, the biology of elements from molecules to the biosphere. Princeton, NJ: Princeton University Press, 439 pp.

[ece35178-bib-0053] Tian, W. , Zhang, H. , Zhao, L. , Zhang, F. , & Huang, H. (2017). Phytoplankton diversity effects on community biomass and stability along nutrient gradients in a Eutrophic Lake. International Journal of Environmental Research and Public Health, 14, 95. 10.3390/ijerph14010095 PMC5295345 28117684

[ece35178-bib-0054] Tilman, D. (1982). Resource competition and community structure. Princeton, NJ: Princeton University Press, 296 pp. 7162524

[ece35178-bib-0055] Tomas, C. R. (1997). Identifying marine phytoplankton. Toronto, ON: Academic Press, Harcourt Brace and Company, 858 pp.

[ece35178-bib-0056] Trimbee, A. M. , & Prepas, E. E. (1987). Evaluation of total phosphorus as a predictor of the relative biomass of blue–green algae with emphasis on Alberta lakes. Canadian Journal of Fisheries and Aquatic Science, 44, 1337–1342.

[ece35178-bib-0057] Truong, C. H. (2012). Shrimp supply chains, common property and pollution management at Tam Giang Cau Hai lagoon, Vietnam, PhD thesis. Lincoln University, Lincoln, New Zealand, 176 pp.

[ece35178-bib-0058] Vallina, S. M. , Follows, M. J. , Dutkiewicz, S. , Montoya, J. M. , Cermeno, P. , & Loreau, M. (2014). Global relationship between phytoplankton diversity and productivity in the ocean. Nature Communications, 5, 4299. 10.1038/ncomms5299 PMC4102128 24980772

[ece35178-bib-0060] Warner, M. E. , Fitt, W. K. , & Schmidt, G. W. (1999). Damage to photosystem II in symbiotic dinoflagellates: A determinant of coral bleaching. Proceedings of the National Academy of Sciences of the USA, 96(14), 8007–8012. 10.1073/pnas.96.14.8007 10393938 PMC22178

[ece35178-bib-0061] Watson, S. B. , McCauley, E. , & Downing, J. A. (1997). Patterns in phytoplankton taxonomic composition across temperate lakes of differing nutrient status. Limnology and Oceanography, 42, 487–495.

[ece35178-bib-0062] Watson, S. B. , Ridal, J. , & Boyer, G. L. (2008). Taste and odour and cyanobacterial toxins: Impairment, prediction, and management in the Great Lakes. Canadian Journal of Fisheries and Aquatic Science, 65, 1779–1796.

[ece35178-bib-0063] West, J. B. , Bowen, G. J. , Cerling, T. E. , & Ehleringer, J. R. (2006). Stable isotopes as one of nature's ecological recorders. Trends in Ecology and Evolution, 21, 408–414. 16753238 10.1016/j.tree.2006.04.002

[ece35178-bib-0064] Wright, D. H. (1983). Species energy theory – an extension of species area theory. Oikos, 41, 496–506.

