# Supplementary material for: Effects of nutrient supply and nutrient ratio on diversity–productivity relationships of phytoplankton in the Cau Hai lagoon, Vietnam
Source: Ecol Evol. 2019 Apr 25;9(10):5950–62. doi: 10.1002/ece3.5178 (PMC6540837; doi:10.1002/ece3.5178)

**Appendices**

**Appendix S1.** Structural equation models of phytoplankton communities from previous field studies (PAR: photosynthetfically active radiation). Blue lines indicate positive paths and red lines indicate negative paths.


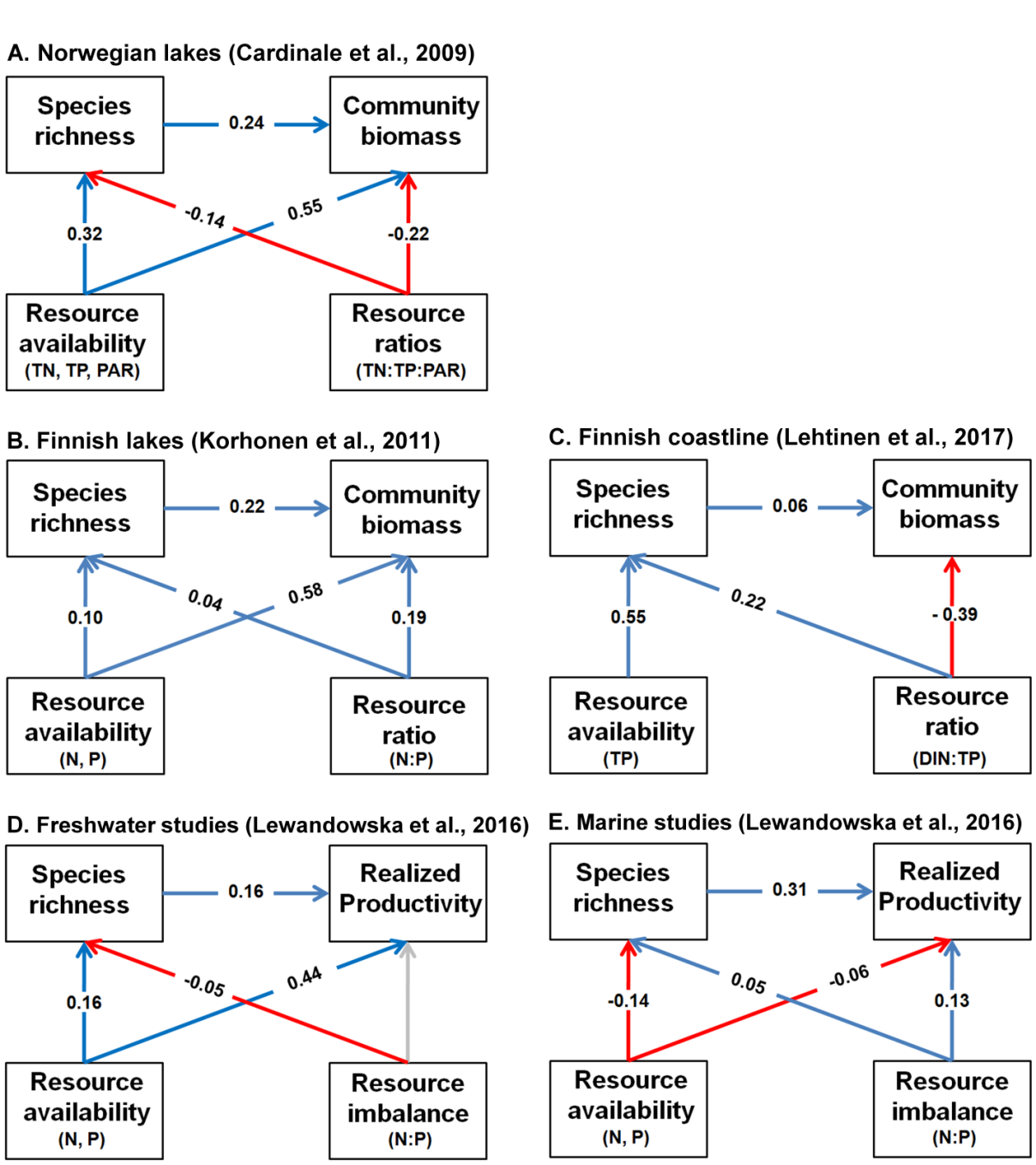
**Appendix S2.** R code of multiple regression analysis of Table 1.

> library(nlme)

>model <- lme(Abundance ~ Temperature + Turbidity + Salinity + TP + DIN + N:P_seston_ + TN + TN:TP + DIN:TP , random = ~ Time | Site, data=data)

>summary(model)

>model <- lme(Richness ~ Temperature + Turbidity + Salinity + TP + DIN + N:P_seston_ + TN + TN:TP + DIN:TP , random = ~ Time | Site, data=data)

>summary(model)

**Appendix S3.** Principal component analysis (PCA) of phytoplankton abundance and richness and environmental variables. Blue dots indicate sampling sites. TotalAbun, TotalR: abundance and species richness of the whole phytoplankton community; CyaAbun, CyaR: abundance and species richness of cyanobacteria; DiaAbun, DiaR: abundance and species richness of diatoms; DinoAbun, DinoR abundance and species richness of dinoflagellates; Temp: temperature; Turb: turbidity; Sal: salinity, DIN:TP: in the water; TN:TP: in the water; N:P: in the seston.


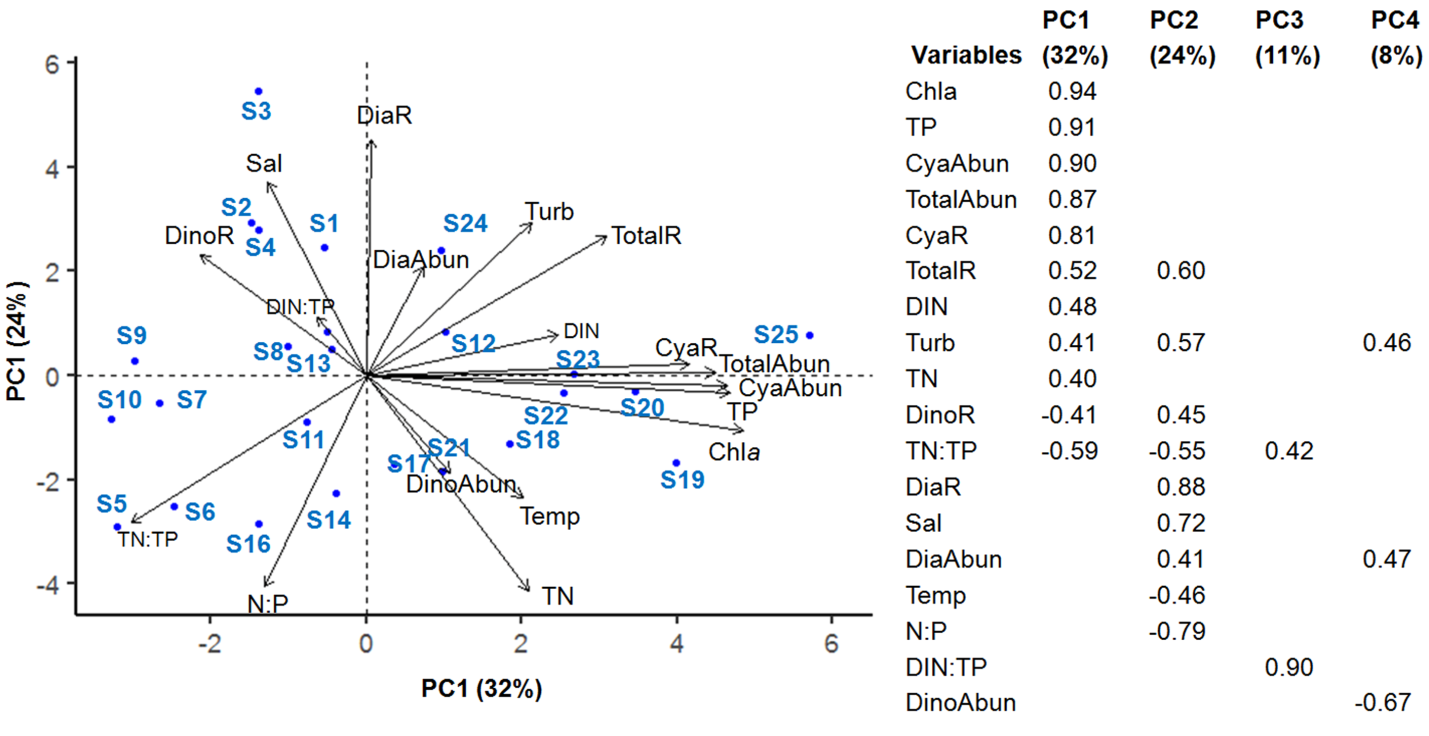


**Appendix S4**. (A) Cluster analysis for the spatial distribution of biotic and abiotic variables (the same variables from Principal component analysis in Appendix S2); (B-F) spatial distribution of TP and DIN concentrations in water, N:P in seston, as well as abundance and richness of phytoplankton in Cau Hai lagoon. Bars show means values.
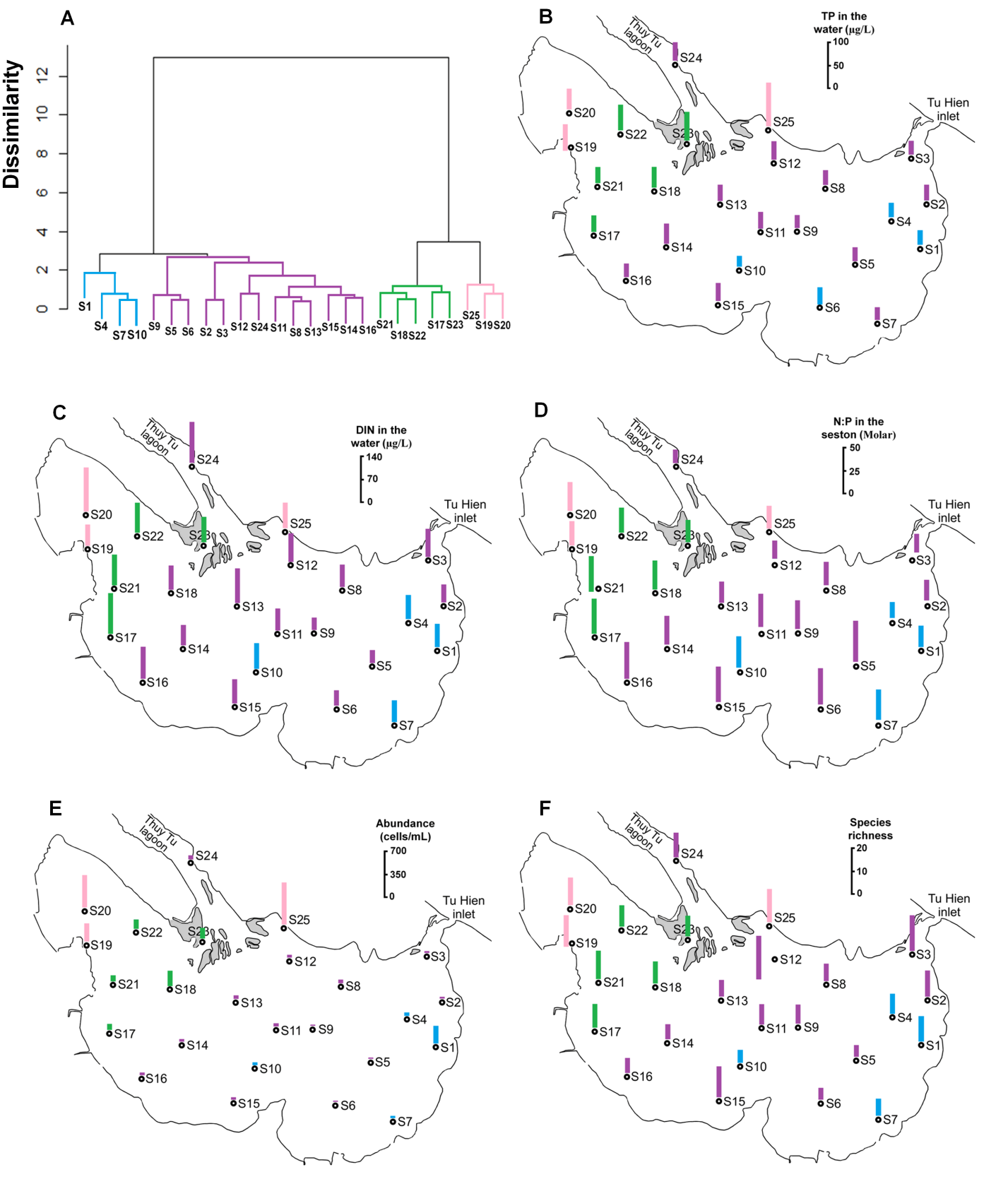


**Appendix S5.** R code and outputs of Structural equation models analysis for Figure 3 in the main text.

> library (lavaan.survey)

> library(semPlot)

***R code for Fig.3a,d,g,k***

> data$TNmean<-mean(data$TN)

> data$TNsd<-sd(data$TN)

> data$TPmean<-mean(data$TP)

> data$TPsd<-sd(data$TP)

> data$TN0<-(data$TN-data$TNmean)/data$TNsd

> data$TP0<-(data$TP-data$TPmean)/data$TPsd

> data$r<- sqrt( data$TN0^2 + data$TP0^2)

> data$y=sqrt(2)

> data$ry<- (data$TN0*1 + data$TP0*1)

> data$a<- data$ry/data$y

> a = ~ data$TN + data$TP

> data$b<- sqrt(data$r^2 - data$a^2)

> A<-'

+ Richness ~ a + b

+ Abundance ~ Richness + a + b

+ Richness ~~ Richness

+ Abundance ~~ Abundance

+ a ~ 0*b'

> fit.data <- sem(A, data = data, fixed.x = T, estimator = "MLM")

> des.data <- svydesign(ids = ~Site, random=~Time, probs = ~1, data = data)

> fit.data.survey <- lavaan.survey(fit.data, des.data, estimator = "MLM")

> summary(fit.data.survey, fit.measures=TRUE)

> semPaths(fit.data.survey, "std", fade=FALSE)

***R code for Fig.3b, e, h, l***

> data$DINmean<-mean(data$DIN)

> data$DINsd<-sd(data$DIN)

> data$TPmean<-mean(data$TP)

> data$TPsd<-sd(data$TP)

> data$DIN0<-(data$DIN-data$DINmean)/data$DINsd

> data$TP0<-(data$TP-data$TPmean)/data$TPsd

> data$r<- sqrt( data$DIN0^2 + data$TP0^2)

> data$y=sqrt(2)

> data$ry<- (data$DIN0*1 + data$TP0*1)

> data$a<- data$ry/data$y

> a = ~ data$DIN + data$TP

> data$b<- sqrt(data$r^2 - data$a^2)

> A<-'

+ Richness ~ a + b

+ Abundance ~ Richness + a + b

+ Richness ~~ Richness

+ Abundance ~~ Abundance

+ a ~ 0*b'

> fit.data <- sem(A, data = data, fixed.x = T, estimator = "MLM")

> des.data <- svydesign(ids = ~Site, random=~Time, probs = ~1, data = data)

> fit.data.survey <- lavaan.survey(fit.data, des.data, estimator = "MLM")

> summary(fit.data.survey, fit.measures=TRUE)

> semPaths(fit.data.survey, "std", fade=FALSE)

***R code for Fig.3c, f, i, m***

> data$DINmean<-mean(data$DIN)

> data$DINsd<-sd(data$DIN)

> data$TPmean<-mean(data$TP)

> data$TPsd<-sd(data$TP)

> data$DIN0<-(data$DIN-data$DINmean)/data$DINsd

> data$TP0<-(data$TP-data$TPmean)/data$TPsd

> data$r<- sqrt( data$DIN0^2 + data$TP0^2)

> data$y=sqrt(2)

> data$ry<- (data$DIN0*1 + data$TP0*1)

> data$a<- data$ry/data$y

> a = ~ data$DIN + data$TP

> A<-'

+ Richness ~ a + NP

+ Abundance ~ Richness + a + NP

+ Richness ~~ Richness

+ Abundance ~~ Abundance

+ a ~ 0*NP'

> fit.data <- sem(A, data = data, fixed.x = T, estimator = "MLM")

> des.data <- svydesign(ids = ~Site, random=~Time, probs = ~1, data = data)

> fit.data.survey <- lavaan.survey(fit.data, des.data, estimator = "MLM")

> summary(fit.data.survey, fit.measures=TRUE)

> semPaths(fit.data.survey, "std", fade=FALSE)

**A. The outputs of the whole phytoplankton community (Fig. 3a)**

lavaan 0.6-3 ended normally after 27 iterations

Optimization method NLMINB

Number of free parameters 11

Number of observations 75

Estimator ML Robust

Model Fit Test Statistic 0.432 0.128

Degrees of freedom 1 1

P-value (Chi-square) 0.511 0.720

Scaling correction factor 3.372

for the Satorra-Bentler correction

Model test baseline model:

Minimum Function Test Statistic 25.949 14.855

Degrees of freedom 6 6

P-value 0.000 0.021

User model versus baseline model:

Comparative Fit Index (CFI) 1.000 1.000

Tucker-Lewis Index (TLI) 1.171 1.591

Robust Comparative Fit Index (CFI) 1.000

Robust Tucker-Lewis Index (TLI) 2.141

Loglikelihood and Information Criteria:

Loglikelihood user model (H0) -217.700 -217.700

Loglikelihood unrestricted model (H1) -217.484 -217.484

Number of free parameters 11 11

Akaike (AIC) 457.400 457.400

Bayesian (BIC) 482.892 482.892

Sample-size adjusted Bayesian (BIC) 448.223 448.223

Root Mean Square Error of Approximation:

RMSEA 0.000 0.000

90 Percent Confidence Interval 0.000 0.264 0.000 0.000

P-value RMSEA <= 0.05 0.549 0.968

Robust RMSEA 0.000

90 Percent Confidence Interval 0.000 0.000

Standardized Root Mean Square Residual:

SRMR 0.022 0.022

Parameter Estimates:

Information Expected

Information saturated (h1) model Structured

Standard Errors Robust.sem

Regressions:

Estimate Std.Err z-value P(>|z|)

Richness ~

a 0.026 0.019 1.374 0.169

b 0.033 0.055 0.589 0.556

Abundance ~

Richness 0.915 0.284 3.221 0.001

a 0.212 0.063 3.343 0.001

b -0.116 0.143 -0.810 0.418

a ~

b 0.000

Intercepts:

Estimate Std.Err z-value P(>|z|)

.Richness 0.956 0.044 21.976 0.000

.Abundance 0.795 0.277 2.876 0.004

.a 0.000 0.204 0.000 1.000

Variances:

Estimate Std.Err z-value P(>|z|)

.Richness 0.043 0.005 8.410 0.000

.Abundance 0.307 0.042 7.343 0.000

.a 1.368 0.325 4.213 0.000

**B. The outputs of the whole phytoplankton community (Fig. 3b)**

lavaan 0.6-3 ended normally after 25 iterations

Optimization method NLMINB

Number of free parameters 11

Number of observations 75

Estimator ML Robust

Model Fit Test Statistic 1.130 0.311

Degrees of freedom 1 1

P-value (Chi-square) 0.288 0.577

Scaling correction factor 3.635

for the Satorra-Bentler correction

Model test baseline model:

Minimum Function Test Statistic 39.036 23.082

Degrees of freedom 6 6

P-value 0.000 0.001

User model versus baseline model:

Comparative Fit Index (CFI) 0.996 1.000

Tucker-Lewis Index (TLI) 0.976 1.242

Robust Comparative Fit Index (CFI) 1.000

Robust Tucker-Lewis Index (TLI) 1.520

Loglikelihood and Information Criteria:

Loglikelihood user model (H0) -214.375 -214.375

Loglikelihood unrestricted model (H1) -213.810 -213.810

Number of free parameters 11 11

Akaike (AIC) 450.749 450.749

Bayesian (BIC) 476.242 476.242

Sample-size adjusted Bayesian (BIC) 441.572 441.572

Root Mean Square Error of Approximation:

RMSEA 0.042 0.000

90 Percent Confidence Interval 0.000 0.313 0.000 0.098

P-value RMSEA <= 0.05 0.332 0.889

Robust RMSEA 0.000

90 Percent Confidence Interval 0.000 0.478

Standardized Root Mean Square Residual:

SRMR 0.039 0.039

Parameter Estimates:

Information Expected

Information saturated (h1) model Structured

Standard Errors Robust.sem

Regressions:

Estimate Std.Err z-value P(>|z|)

Richness ~

a 0.058 0.017 3.331 0.001

b -0.020 0.045 -0.457 0.648

Abundance ~

Richness 0.710 0.268 2.647 0.008

a 0.233 0.045 5.236 0.000

b 0.272 0.100 2.728 0.086

a ~

b 0.000

Intercepts:

Estimate Std.Err z-value P(>|z|)

.Richness 0.991 0.039 25.414 0.000

.Abundance 0.734 0.268 2.739 0.006

.a -0.000 0.182 -0.000 1.000

Variances:

Estimate Std.Err z-value P(>|z|)

.Richness 0.040 0.005 8.515 0.000

.Abundance 0.279 0.041 6.769 0.000

.a 1.241 0.242 5.132 0.000

**C. The outputs of the whole phytoplankton community (Fig. 3c)**

lavaan 0.6-3 ended normally after 33 iterations

Optimization method NLMINB

Number of free parameters 11

Number of observations 75

Estimator ML Robust

Model Fit Test Statistic 3.746 1.138

Degrees of freedom 1 1

P-value (Chi-square) 0.053 0.286

Scaling correction factor 3.293

for the Satorra-Bentler correction

Model test baseline model:

Minimum Function Test Statistic 53.160 35.871

Degrees of freedom 6 6

P-value 0.000 0.000

User model versus baseline model:

Comparative Fit Index (CFI) 0.942 0.995

Tucker-Lewis Index (TLI) 0.651 0.972

Robust Comparative Fit Index (CFI) 0.990

Robust Tucker-Lewis Index (TLI) 0.939

Loglikelihood and Information Criteria:

Loglikelihood user model (H0) -133.655 -133.655

Loglikelihood unrestricted model (H1) -131.782 -131.782

Number of free parameters 11 11

Akaike (AIC) 289.310 289.310

Bayesian (BIC) 314.802 314.802

Sample-size adjusted Bayesian (BIC) 280.133 280.133

Root Mean Square Error of Approximation:

RMSEA 0.191 0.043

90 Percent Confidence Interval 0.000 0.413 0.000 0.197

P-value RMSEA <= 0.05 0.075 0.420

Robust RMSEA 0.078

90 Percent Confidence Interval 0.000 0.568

Standardized Root Mean Square Residual:

SRMR 0.073 0.073

Parameter Estimates:

Information Expected

Information saturated (h1) model Structured

Standard Errors Robust.sem

Regressions:

Estimate Std.Err z-value P(>|z|)

Richness ~

a 0.040 0.015 2.662 0.008

NP -0.442 0.114 -3.881 0.000

Abundance ~

Richness 0.928 0.360 2.575 0.010

a 0.262 0.049 5.311 0.000

NP 0.698 0.387 1.801 0.072

a ~

NP 0.000

Intercepts:

Estimate Std.Err z-value P(>|z|)

.Richness 1.612 0.165 9.749 0.000

.Abundance -0.292 0.832 -0.350 0.726

.a -0.000 0.182 -0.000 1.000

Variances:

Estimate Std.Err z-value P(>|z|)

.Richness 0.034 0.004 8.175 0.000

.Abundance 0.284 0.044 6.496 0.000

.a 1.241 0.242 5.132 0.000

**D. The outputs of cyanobacteria (Fig. 3d)**

lavaan 0.6-3 ended normally after 32 iterations

Optimization method NLMINB

Number of free parameters 11

Number of observations 50

Estimator ML Robust

Model Fit Test Statistic 0.001 0.001

Degrees of freedom 1 1

P-value (Chi-square) 0.994 0.996

Scaling correction factor 3.091

for the Satorra-Bentler correction

Model test baseline model:

Minimum Function Test Statistic 38.191 23.678

Degrees of freedom 6 6

P-value 0.000 0.001

User model versus baseline model:

Comparative Fit Index (CFI) 1.000 1.000

Tucker-Lewis Index (TLI) 1.186 1.339

Robust Comparative Fit Index (CFI) 1.000

Robust Tucker-Lewis Index (TLI) 1.650

Loglikelihood and Information Criteria:

Loglikelihood user model (H0) -149.258 -149.258

Loglikelihood unrestricted model (H1) -149.258 -149.258

Number of free parameters 11 11

Akaike (AIC) 320.516 320.516

Bayesian (BIC) 341.548 341.548

Sample-size adjusted Bayesian (BIC) 307.021 307.021

Root Mean Square Error of Approximation:

RMSEA 0.000 0.000

90 Percent Confidence Interval 0.000 0.000 0.000 0.000

P-value RMSEA <= 0.05 0.994 1.000

Robust RMSEA 0.000

90 Percent Confidence Interval 0.000 0.000

Standardized Root Mean Square Residual:

SRMR 0.000 0.000

Parameter Estimates:

Information Expected

Information saturated (h1) model Structured

Standard Errors Robust.sem

Regressions:

Estimate Std.Err z-value P(>|z|)

Richness ~

a 0.092 0.014 6.538 0.017

b 0.027 0.072 0.374 0.709

Abundance ~

Richness 0.227 0.538 0.422 0.673

a 0.467 0.105 4.428 0.000

b -0.069 0.216 -0.317 0.751

a ~

b 0.000

Intercepts:

Estimate Std.Err z-value P(>|z|)

.Richness 0.512 0.057 9.002 0.000

.Abundance 1.061 0.308 3.444 0.001

.a -0.000 0.229 -0.000 1.000

Variances:

Estimate Std.Err z-value P(>|z|)

.Richness 0.037 0.010 3.570 0.000

.Abundance 0.516 0.064 8.093 0.000

.a 1.365 0.356 3.834 0.000

**E. The outputs of cyanobacteria (Fig. 3e)**

lavaan 0.6-3 ended normally after 24 iterations

Optimization method NLMINB

Number of free parameters 11

Number of observations 50

Estimator ML Robust

Model Fit Test Statistic 0.729 0.284

Degrees of freedom 1 1

P-value (Chi-square) 0.393 0.594

Scaling correction factor 2.566

for the Satorra-Bentler correction

Model test baseline model:

Minimum Function Test Statistic 28.937 17.038

Degrees of freedom 6 6

P-value 0.000 0.009

User model versus baseline model:

Comparative Fit Index (CFI) 1.000 1.000

Tucker-Lewis Index (TLI) 1.071 1.389

Robust Comparative Fit Index (CFI) 1.000

Robust Tucker-Lewis Index (TLI) 1.588

Loglikelihood and Information Criteria:

Loglikelihood user model (H0) -159.512 -159.512

Loglikelihood unrestricted model (H1) -159.148 -159.148

Number of free parameters 11 11

Akaike (AIC) 341.025 341.025

Bayesian (BIC) 362.057 362.057

Sample-size adjusted Bayesian (BIC) 327.530 327.530

Root Mean Square Error of Approximation:

RMSEA 0.000 0.000

90 Percent Confidence Interval 0.000 0.353 0.000 0.158

P-value RMSEA <= 0.05 0.422 0.830

Robust RMSEA 0.000

90 Percent Confidence Interval 0.000 0.485

Standardized Root Mean Square Residual:

SRMR 0.039 0.039

Parameter Estimates:

Information Expected

Information saturated (h1) model Structured

Standard Errors Robust.sem

Regressions:

Estimate Std.Err z-value P(>|z|)

Richness ~

a 0.061 0.024 2.530 0.011

b 0.027 0.069 0.393 0.695

Abundance ~

Richness 0.740 0.602 1.230 0.219

a 0.435 0.072 6.055 0.000

b -0.033 0.210 -0.158 0.874

a ~

b 0.000

Intercepts:

Estimate Std.Err z-value P(>|z|)

.Richness 0.511 0.058 8.790 0.000

.Abundance 0.768 0.294 2.611 0.009

.a -0.000 0.214 -0.000 1.000

Variances:

Estimate Std.Err z-value P(>|z|)

.Richness 0.044 0.009 4.751 0.000

.Abundance 0.534 0.104 5.127 0.000

.a 1.245 0.312 3.984 0.000

**F. The outputs of cyanobacteria (Fig. 3f)**

lavaan 0.6-3 ended normally after 29 iterations

Optimization method NLMINB

Number of free parameters 11

Number of observations 50

Estimator ML Robust

Model Fit Test Statistic 2.797 1.048

Degrees of freedom 1 1

P-value (Chi-square) 0.094 0.306

Scaling correction factor 2.668

for the Satorra-Bentler correction

Model test baseline model:

Minimum Function Test Statistic 32.534 20.952

Degrees of freedom 6 6

P-value 0.000 0.002

User model versus baseline model:

Comparative Fit Index (CFI) 0.932 0.997

Tucker-Lewis Index (TLI) 0.594 0.981

Robust Comparative Fit Index (CFI) 0.994

Robust Tucker-Lewis Index (TLI) 0.967

Loglikelihood and Information Criteria:

Loglikelihood user model (H0) -109.720 -109.720

Loglikelihood unrestricted model (H1) -108.321 -108.321

Number of free parameters 11 11

Akaike (AIC) 241.439 241.439

Bayesian (BIC) 262.472 262.472

Sample-size adjusted Bayesian (BIC) 227.945 227.945

Root Mean Square Error of Approximation:

RMSEA 0.190 0.031

90 Percent Confidence Interval 0.000 0.469 0.000 0.254

P-value RMSEA <= 0.05 0.115 0.417

Robust RMSEA 0.051

90 Percent Confidence Interval 0.000 0.616

Standardized Root Mean Square Residual:

SRMR 0.077 0.077

Parameter Estimates:

Information Expected

Information saturated (h1) model Structured

Standard Errors Robust.sem

Regressions:

Estimate Std.Err z-value P(>|z|)

Richness ~

a 0.060 0.024 2.454 0.014

NP -0.057 0.182 -0.312 0.755

Abundance ~

Richness 0.765 0.540 1.417 0.157

a 0.460 0.067 6.847 0.000

NP 0.739 0.390 1.895 0.058

a ~

NP 0.000

Intercepts:

Estimate Std.Err z-value P(>|z|)

.Richness 0.610 0.260 2.350 0.019

.Abundance -0.313 0.575 -0.544 0.587

.a -0.000 0.214 -0.000 1.000

Variances:

Estimate Std.Err z-value P(>|z|)

.Richness 0.044 0.009 4.998 0.000

.Abundance 0.517 0.102 5.063 0.000

.a 1.245 0.312 3.984 0.000

**G. The outputs of diatoms (Fig. 3g)**

lavaan 0.6-3 ended normally after 25 iterations

Optimization method NLMINB

Number of free parameters 11

Number of observations 74

Estimator ML Robust

Model Fit Test Statistic 0.782 0.215

Degrees of freedom 1 1

P-value (Chi-square) 0.376 0.643

Scaling correction factor 3.639

for the Satorra-Bentler correction

Model test baseline model:

Minimum Function Test Statistic 18.000 11.605

Degrees of freedom 6 6

P-value 0.006 0.071

User model versus baseline model:

Comparative Fit Index (CFI) 1.000 1.000

Tucker-Lewis Index (TLI) 1.109 1.840

Robust Comparative Fit Index (CFI) 1.000

Robust Tucker-Lewis Index (TLI) 2.972

Loglikelihood and Information Criteria:

Loglikelihood user model (H0) -223.631 -223.631

Loglikelihood unrestricted model (H1) -223.240 -223.240

Number of free parameters 11 11

Akaike (AIC) 469.261 469.261

Bayesian (BIC) 494.606 494.606

Sample-size adjusted Bayesian (BIC) 459.941 459.941

Root Mean Square Error of Approximation:

RMSEA 0.000 0.000

90 Percent Confidence Interval 0.000 0.294 0.000 0.066

P-value RMSEA <= 0.05 0.419 0.938

Robust RMSEA 0.000

90 Percent Confidence Interval 0.000 0.455

Standardized Root Mean Square Residual:

SRMR 0.028 0.028

Parameter Estimates:

Information Expected

Information saturated (h1) model Structured

Standard Errors Robust.sem

Regressions:

Estimate Std.Err z-value P(>|z|)

Richness ~

a -0.041 0.024 -1.681 0.093

b -0.014 0.064 -0.224 0.823

Abundance ~

Richness 1.041 0.291 3.581 0.000

a -0.020 0.078 -0.255 0.799

b 0.037 0.094 0.393 0.694

a ~

b 0.000

Intercepts:

Estimate Std.Err z-value P(>|z|)

.Richness 0.618 0.046 13.308 0.000

.Abundance 0.169 0.192 0.880 0.379

.a 0.000 0.207 0.000 1.000

Variances:

Estimate Std.Err z-value P(>|z|)

.Richness 0.054 0.006 9.702 0.000

.Abundance 0.308 0.056 5.485 0.000

.a 1.387 0.328 4.231 0.000

**H. The outputs of diatoms (Fig. 3h)**

lavaan 0.6-3 ended normally after 25 iterations

Optimization method NLMINB

Number of free parameters 11

Number of observations 74

Estimator ML Robust

Model Fit Test Statistic 1.153 0.323

Degrees of freedom 1 1

P-value (Chi-square) 0.283 0.570

Scaling correction factor 3.571

for the Satorra-Bentler correction

Model test baseline model:

Minimum Function Test Statistic 20.107 15.363

Degrees of freedom 6 6

P-value 0.003 0.018

User model versus baseline model:

Comparative Fit Index (CFI) 0.989 1.000

Tucker-Lewis Index (TLI) 0.935 1.434

Robust Comparative Fit Index (CFI) 1.000

Robust Tucker-Lewis Index (TLI) 2.184

Loglikelihood and Information Criteria:

Loglikelihood user model (H0) -225.398 -225.398

Loglikelihood unrestricted model (H1) -224.822 -224.822

Number of free parameters 11 11

Akaike (AIC) 472.796 472.796

Bayesian (BIC) 498.141 498.141

Sample-size adjusted Bayesian (BIC) 463.476 463.476

Root Mean Square Error of Approximation:

RMSEA 0.045 0.000

90 Percent Confidence Interval 0.000 0.316 0.000 0.104

P-value RMSEA <= 0.05 0.326 0.879

Robust RMSEA 0.000

90 Percent Confidence Interval 0.000 0.480

Standardized Root Mean Square Residual:

SRMR 0.034 0.034

Parameter Estimates:

Information Expected

Information saturated (h1) model Structured

Standard Errors Robust.sem

Regressions:

Estimate Std.Err z-value P(>|z|)

Richness ~

a 0.015 0.017 0.862 0.389

b -0.012 0.052 -0.237 0.812

Abundance ~

Richness 1.054 0.254 4.142 0.000

a 0.043 0.050 0.870 0.384

b 0.246 0.128 1.924 0.054

a ~

b 0.000

Intercepts:

Estimate Std.Err z-value P(>|z|)

.Richness 0.618 0.045 13.652 0.000

.Abundance 0.015 0.215 0.069 0.945

.a -0.000 0.184 -0.000 1.000

Variances:

Estimate Std.Err z-value P(>|z|)

.Richness 0.056 0.007 7.996 0.000

.Abundance 0.289 0.046 6.251 0.000

.a 1.243 0.243 5.110 0.000

**I. The outputs of diatoms (Fig. 3i)**

lavaan 0.6-3 ended normally after 31 iterations

Optimization method NLMINB

Number of free parameters 11

Number of observations 74

Estimator ML Robust

Model Fit Test Statistic 3.621 1.110

Degrees of freedom 1 1

P-value (Chi-square) 0.057 0.292

Scaling correction factor 3.261

for the Satorra-Bentler correction

Model test baseline model:

Minimum Function Test Statistic 30.022 25.495

Degrees of freedom 6 6

P-value 0.000 0.000

User model versus baseline model:

Comparative Fit Index (CFI) 0.891 0.994

Tucker-Lewis Index (TLI) 0.345 0.966

Robust Comparative Fit Index (CFI) 0.984

Robust Tucker-Lewis Index (TLI) 0.906

Loglikelihood and Information Criteria:

Loglikelihood user model (H0) -147.675 -147.675

Loglikelihood unrestricted model (H1) -145.865 -145.865

Number of free parameters 11 11

Akaike (AIC) 317.351 317.351

Bayesian (BIC) 342.695 342.695

Sample-size adjusted Bayesian (BIC) 308.030 308.030

Root Mean Square Error of Approximation:

RMSEA 0.188 0.039

90 Percent Confidence Interval 0.000 0.412 0.000 0.198

P-value RMSEA <= 0.05 0.080 0.430

Robust RMSEA 0.070

90 Percent Confidence Interval 0.000 0.566

Standardized Root Mean Square Residual:

SRMR 0.064 0.064

Parameter Estimates:

Information Expected

Information saturated (h1) model Structured

Standard Errors Robust.sem

Regressions:

Estimate Std.Err z-value P(>|z|)

Richness ~

a -0.003 0.016 -0.215 0.830

NP -0.476 0.099 -4.791 0.000

Abundance ~

Richness 0.955 0.285 3.344 0.001

a 0.047 0.050 0.946 0.344

NP -0.303 0.454 -0.669 0.504

a ~

NP 0.000

Intercepts:

Estimate Std.Err z-value P(>|z|)

.Richness 1.292 0.144 8.960 0.000

.Abundance 0.679 0.735 0.924 0.355

.a -0.000 0.184 -0.000 1.000

Variances:

Estimate Std.Err z-value P(>|z|)

.Richness 0.049 0.006 7.613 0.000

.Abundance 0.302 0.054 5.548 0.000

.a 1.243 0.243 5.110 0.000

**K. The outputs of dinoflagellates (Fig. 3k)**

lavaan 0.6-3 ended normally after 31 iterations

Optimization method NLMINB

Number of free parameters 11

Number of observations 74

Estimator ML Robust

Model Fit Test Statistic 0.800 0.221

Degrees of freedom 1 1

P-value (Chi-square) 0.371 0.638

Scaling correction factor 3.616

for the Satorra-Bentler correction

Model test baseline model:

Minimum Function Test Statistic 1.498 1.067

Degrees of freedom 6 6

P-value 0.960 0.983

User model versus baseline model:

Comparative Fit Index (CFI) 1.000 1.000

Tucker-Lewis Index (TLI) 0.734 0.053

Robust Comparative Fit Index (CFI) 1.000

Robust Tucker-Lewis Index (TLI) -1.438

Loglikelihood and Information Criteria:

Loglikelihood user model (H0) -186.816 -186.816

Loglikelihood unrestricted model (H1) -186.416 -186.416

Number of free parameters 11 11

Akaike (AIC) 395.633 395.633

Bayesian (BIC) 420.978 420.978

Sample-size adjusted Bayesian (BIC) 386.312 386.312

Root Mean Square Error of Approximation:

RMSEA 0.000 0.000

90 Percent Confidence Interval 0.000 0.295 0.000 0.070

P-value RMSEA <= 0.05 0.414 0.934

Robust RMSEA 0.000

90 Percent Confidence Interval 0.000 0.456

Standardized Root Mean Square Residual:

SRMR 0.028 0.028

Parameter Estimates:

Information Expected

Information saturated (h1) model Structured

Standard Errors Robust.sem

Regressions:

Estimate Std.Err z-value P(>|z|)

Richness ~

a -0.003 0.012 -0.256 0.798

b 0.011 0.037 0.308 0.758

Abundance ~

Richness -0.138 0.348 -0.398 0.691

a 0.034 0.053 0.637 0.524

b 0.012 0.151 0.077 0.939

a ~

b 0.000

Intercepts:

Estimate Std.Err z-value P(>|z|)

.Richness 0.529 0.029 18.411 0.000

.Abundance 1.131 0.224 5.049 0.000

.a 0.000 0.206 0.000 1.000

Variances:

Estimate Std.Err z-value P(>|z|)

.Richness 0.023 0.004 5.953 0.000

.Abundance 0.270 0.033 8.240 0.000

.a 1.379 0.327 4.221 0.000

**L. The outputs of dinoflagellates (Fig. 3l)**

lavaan 0.6-3 ended normally after 32 iterations

Optimization method NLMINB

Number of free parameters 11

Number of observations 74

Estimator ML Robust

Model Fit Test Statistic 1.981 0.557

Degrees of freedom 1 1

P-value (Chi-square) 0.159 0.456

Scaling correction factor 3.557

for the Satorra-Bentler correction

Model test baseline model:

Minimum Function Test Statistic 6.187 4.689

Degrees of freedom 6 6

P-value 0.403 0.584

User model versus baseline model:

Comparative Fit Index (CFI) 0.000 1.000

Tucker-Lewis Index (TLI) -30.417 -1.027

Robust Comparative Fit Index (CFI) 1.000

Robust Tucker-Lewis Index (TLI) -4.465

Loglikelihood and Information Criteria:

Loglikelihood user model (H0) -186.981 -186.981

Loglikelihood unrestricted model (H1) -185.991 -185.991

Number of free parameters 11 11

Akaike (AIC) 395.962 395.962

Bayesian (BIC) 421.307 421.307

Sample-size adjusted Bayesian (BIC) 386.642 386.642

Root Mean Square Error of Approximation:

RMSEA 0.115 0.000

90 Percent Confidence Interval 0.000 0.355 0.000 0.142

P-value RMSEA <= 0.05 0.197 0.739

Robust RMSEA 0.000

90 Percent Confidence Interval 0.000 0.522

Standardized Root Mean Square Residual:

SRMR 0.045 0.045

Parameter Estimates:

Information Expected

Information saturated (h1) model Structured

Standard Errors Robust.sem

Regressions:

Estimate Std.Err z-value P(>|z|)

Richness ~

a -0.003 0.016 -0.212 0.832

b -0.021 0.037 -0.564 0.573

Abundance ~

Richness -0.084 0.361 -0.234 0.815

a 0.048 0.059 0.818 0.414

b 0.182 0.112 1.628 0.104

a ~

b 0.000

Intercepts:

Estimate Std.Err z-value P(>|z|)

.Richness 0.550 0.031 17.590 0.000

.Abundance 0.980 0.241 4.065 0.000

.a -0.000 0.179 -0.000 1.000

Variances:

Estimate Std.Err z-value P(>|z|)

.Richness 0.023 0.004 6.092 0.000

.Abundance 0.259 0.034 7.641 0.000

.a 1.216 0.232 5.248 0.000

**M. The outputs of dinoflagellates (Fig. 3m)**

lavaan 0.6-3 ended normally after 36 iterations

Optimization method NLMINB

Number of free parameters 11

Number of observations 74

Estimator ML Robust

Model Fit Test Statistic 1.986 0.671

Degrees of freedom 1 1

P-value (Chi-square) 0.159 0.413

Scaling correction factor 2.959

for the Satorra-Bentler correction

Model test baseline model:

Minimum Function Test Statistic 6.707 4.700

Degrees of freedom 6 6

P-value 0.349 0.583

User model versus baseline model:

Comparative Fit Index (CFI) 0.000 1.000

Tucker-Lewis Index (TLI) -7.378 -0.517

Robust Comparative Fit Index (CFI) 1.000

Robust Tucker-Lewis Index (TLI) -2.145

Loglikelihood and Information Criteria:

Loglikelihood user model (H0) -110.806 -110.806

Loglikelihood unrestricted model (H1) -109.812 -109.812

Number of free parameters 11 11

Akaike (AIC) 243.611 243.611

Bayesian (BIC) 268.956 268.956

Sample-size adjusted Bayesian (BIC) 234.291 234.291

Root Mean Square Error of Approximation:

RMSEA 0.115 0.000

90 Percent Confidence Interval 0.000 0.355 0.000 0.170

P-value RMSEA <= 0.05 0.197 0.638

Robust RMSEA 0.000

90 Percent Confidence Interval 0.000 0.492

Standardized Root Mean Square Residual:

SRMR 0.045 0.045

Parameter Estimates:

Information Expected

Information saturated (h1) model Structured

Standard Errors Robust.sem

Regressions:

Estimate Std.Err z-value P(>|z|)

Richness ~

a -0.009 0.016 -0.555 0.579

NP -0.143 0.130 -1.103 0.270

Abundance ~

Richness -0.057 0.324 -0.175 0.861

a 0.071 0.054 1.328 0.184

NP 0.339 0.305 1.114 0.265

a ~

NP 0.000

Intercepts:

Estimate Std.Err z-value P(>|z|)

.Richness 0.741 0.187 3.966 0.000

.Abundance 0.609 0.473 1.288 0.198

.a -0.000 0.179 -0.000 1.000

Variances:

Estimate Std.Err z-value P(>|z|)

.Richness 0.022 0.004 5.888 0.000

.Abundance 0.263 0.033 7.904 0.000

.a 1.216 0.232 5.248 0.000

**Appendix S6.** Summary statistics for water and seston parameters (means ± SE), as well as one-way ANOVA results for the effects (F values) of sampling time and sampling site. The number of observations for each variableis 75. Significant values are in bold and levels of significance are as follows: **p*<0.05; ***p* < 0.01; ****p* < 0.001.

| **Variable** | **Mean ± SE** | **Min** | **Max** | **Source of variation (F value)** | | |
| --- | --- | --- | --- | --- | --- | --- |
|  |  |  |  | **Time effect** | **Site effect** |  |
| Temperature (^0^C) | 30.5 ± 0.3 | 25.4 | 34.6 | **194.6***** | 1.3 |  |
| pH | 8.4± 0.1 | 6.0 | 9.4 | **23.7***** | 0.6 |  |
| DO (mg/L) | 6.6 ± 0.2 | 4.1 | 11.8 | **34.4***** | 0.3 |  |
| Salinity (‰) | 11.4 ± 0.5 | 2.9 | 28.6 | **21.6***** | **13.3***** |  |
| Turbidity (NTU) | 8.6 ± 1.2 | 0.1 | 47.7 | **72.7***** | 0.9 |  |
| Depth (m) | 1.5 ±0.1 | 0.3 | 2.3 | **3.4*** | **15.1*** |  |
| DIN (µg/L) | 76.3±3.5 | 19.1 | 165.6 | 1.4 | 1.9 |  |
| TN (µg/L) | 326.9 ± 10.5 | 150.4 | 660.1 | 0.3 | **4.4***** |  |
| TP (µg/L) | 38.8 ±2.0 | 15.6 | 98.4 | 0.1 | **4.5***** |  |
| DIN:TP | 4.8±0.3 | 1.6 | 13.1 | 0.7 | 1.8 |  |
| TN:TP (Molar) | 20.6 ± 0.8 | 6.3 | 39.3 | 0.01 | 3.0 |  |
| Chl*a* (µg/L) | 6.4 ± 0.7 | 0.7 | 31.0 | 0.9 | **6.5***** |  |
| TSI | 46.3±0.6 | 34.7 | 59.0 | 0.5 | **8.4***** |  |
| C_Seston_ (mg/L) | 1.2 ± 0.1 | 0.2 | 2.5 | 1.8 | **3.1***** |  |
| N_Seston_ (µg/L) | 194 ± 1.0 | 38.5 | 440.0 | 0.9 | **3.1***** |  |
| P_Seston_ (µg/L) | 16.5±1.0 | 2.3 | 58.0 | 0.7 | 0.9 |  |
| C:N_Seston_ (Molar) | 7.4 ± 0.1 | 5.7 | 9.3 | **3.5*** | 0.9 |  |
| C:P_Seston_ (Molar) | 207.7± 8.3 | 64.1 | 388.6 | 1.0 | 1.8 |  |
| N:P_Seston_ (Molar) | 28.6± 1.3 | 7.4 | 62.6 | 0.4 | 1.7 |  |
| δ^15^N_Seston_ (‰) | 8.7 ± 0.3 | 1.1 | 14.2 | **11.5***** | **1.9*** |  |
| Species richness | 9.6 ± 0.6 | 2.0 | 26 | **7.4**** | 1.9 |  |
| Total abundance (cells/mL) | 119.4 ± 22.9 | 1.6 | 825.3 | **5.8**** | 1.9 |  |

**Appendix S7.** Mean proportions of the abundance (A) and species composition (B) of the three functional groups in the Cau Hai lagoon study.


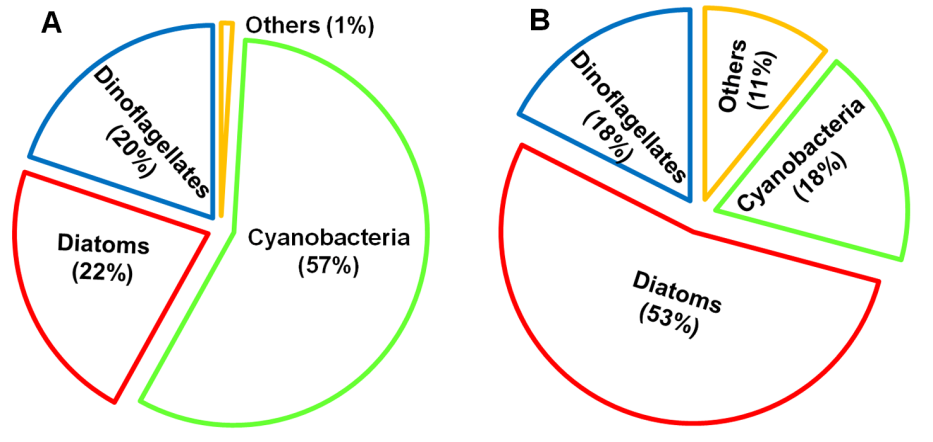

Supplement: Supplementary file 1 [file ECE3-9-5950-s001.docx]
